# Surprising spatiotemporal stability of a multi‐peak fitness landscape revealed by independent field experiments measuring hybrid fitness

**DOI:** 10.1002/evl3.195

**Published:** 2020-10-21

**Authors:** Christopher H. Martin, Katelyn J. Gould

**Affiliations:** ^1^ Department of Integrative Biology University of California, Berkeley Berkeley California 94720; ^2^ Museum of Vertebrate Zoology University of California, Berkeley Berkeley California 94720; ^3^ Department of Biology University of North Carolina at Chapel Hill Chapel Hill North Carolina 27515

**Keywords:** Adaptive dynamics, adaptive radiation, competition, key innovation, lepidophagy, negative frequency‐dependent disruptive selection, novelty, trophic specialization

## Abstract

The effect of the environment on fitness in natural populations is a fundamental question in evolutionary biology. However, experimental manipulations of both environment and phenotype at the same time are rare. Thus, the relative importance of the competitive environment versus intrinsic organismal performance in shaping the location, height, and fluidity of fitness peaks and valleys remains largely unknown. Here, we experimentally tested the effect of competitor frequency on the complex fitness landscape driving adaptive radiation of a generalist and two trophic specialist pupfishes, a scale‐eater and molluscivore, endemic to hypersaline lakes on San Salvador Island (SSI), Bahamas. We manipulated phenotypes, by generating 3407 F4/F5 lab‐reared hybrids, and competitive environment, by altering the frequency of rare transgressive hybrids between field enclosures in two independent lake populations. We then tracked hybrid survival and growth rates across these four field enclosures for 3–11 months. In contrast to competitive speciation theory, we found no evidence that the frequency of hybrid phenotypes affected their survival. Instead, we observed a strikingly similar fitness landscape to a previous independent field experiment, each supporting multiple fitness peaks for generalist and molluscivore phenotypes and a large fitness valley isolating the divergent scale‐eater phenotype. These features of the fitness landscape were stable across manipulated competitive environments, multivariate trait axes, and spatiotemporal heterogeneity. We suggest that absolute performance constraints and divergent gene regulatory networks shape macroevolutionary (interspecific) fitness landscapes in addition to microevolutionary (intraspecific) competitive dynamics. This interplay between organism and environment underlies static and dynamic features of the adaptive landscape.

The adaptive landscape, the complex mapping of fitness onto phenotype or genotype, is both a central unifying concept in evolutionary biology and an empirical measurement (Wright 1932; Lande 1979; Gavrilets 2004; Carneiro and Hartl 2010; Svensson and Calsbeek 2012) that links the microevolutionary processes of natural and sexual selection in wild populations with macroevolutionary patterns of speciation, novelty, and adaptive radiation (Lande and Arnold 1983; Arnold et al. 2001; Kingsolver et al. 2001; Martin and Richards 2019).

Despite its central importance, it remains unclear what factors shape the fitness landscape across space and time. In classical views arising from both Wright's (1932) and Simpson's (1944) original conceptions of genotypic and phenotypic fitness landscapes, respectively, and Fisher's (1930) geometric model, fitness optima occur on a high‐dimensional landscape with both static and dynamic features. Static features of fitness landscapes arise due to negative epistasis within genotypic networks (Whitlock et al. 1995; Weinreich et al. 2005; Wright 1932) and functional trade‐offs between different ecological niches or collections of similar niches known as adaptive zones (Simpson 1944; Roughgarden 1972; Higham et al. 2016; Evans et al. 2017). Dynamic features, emphasized by the more recent paradigm of adaptive dynamics originating in game theory (Abrams et al. 1993; Abrams 2001; Bolnick 2004; Doebeli and Dieckmann 2005), arise because the fitness landscape resembles a trampoline: as the relative frequency of phenotypically similar individuals competing for the same limited resources increases, their fitness decreases due to increased competition, whereas rare phenotypes at the extremes of the phenotype distribution should have a fitness advantage due to reduced competition for additional resources that they are able to exploit (Rosenzweig 1978). This process is known as negative frequency‐dependent disruptive selection and can lead to “competitive” speciation, even when adapting to a unimodal resource distribution (Rosenzweig 1978; Dieckmann and Doebeli 1999; Kirkpatrick and Ravigné 2002; Matessi et al. 2002; Doebeli et al. 2005; Bürger and Schneider 2006; Otto et al. 2008). Laboratory and field studies of natural populations provide extensive support for negative frequency‐dependent disruptive selection (Pfennig 1992; Hori 1993; Sinervo et al. 2000; Schluter 2003; Bolnick 2004; Kassen et al. 2004; Olendorf et al. 2006; Bolnick and Lau 2008; Weeks and Hoffmann 2008; Koskella and Lively 2009; Svensson and Calsbeek 2012; Kusche et al. 2012; Bolnick and Stutz 2017; Nosil et al. 2018), to the extent that some investigators assert its universality in all natural populations (Haller and Hendry 2014). Due to its elegance and mathematical tractability, frequency dependence has also been widely adopted by speciation theorists as the sole mechanism for disruptive selection in most models (Gavrilets 2004; Polechová and Barton 2005; Otto et al. 2008; Servedio and Boughman 2017; Martin and Richards 2019).

However, the relative contributions of the dynamic competitive environment versus stable fitness optima in shaping the broader topography of fitness landscapes across multiple species remain unclear, despite the importance of these interactions in providing the bridge between microevolutionary processes within a population and macroevolutionary scale patterns of diversification and adaptive radiation (Arnold et al. 2001; Higham et al. 2016; Hendry 2017; Martin and Richards 2019; Martin et al. 2019; Gillespie et al. 2020). For example, although negative frequency‐dependent disruptive selection may be ubiquitous within populations, the threshold of phenotypic similarity necessary for intraspecific competition within the same ecological niche is rarely measured and this competition kernel can have major impacts on speciation (Dieckmann and Doebeli 1999; Baptestini et al. 2009). Similarly, shifts in the location of fitness optima due to environmental stochasticity are often assumed to follow a Brownian motion process (Grant and Grant 2002; Hansen et al. 2008) without accounting for hard boundaries imposed by functional trade‐offs or biophysical constraints (but see Enquist and Niklas 2002; Boucher and Demery 2016). Conversely, over broader macroevolutionary timescales the role of stable fitness optima in shaping trait diversification across a radiation is frequently assumed and tested by fitting Ornstein‐Uhlenbeck models of trait diversification using phylogenetic comparative methods (Butler and King 2004; Harmon et al. 2010; Beaulieu and O'Meara 2012; O'Meara 2012; Uyeda and Harmon 2014; Burress and Tan 2017) while not accounting for the effects of density‐ and frequency‐dependent selection due to competition within a community (but see Rabosky 2009; Harmon et al. 2019; Landis et al. 2019).

Previous field experiments testing frequency‐dependent selection have focused on only a single population or pair of ecomorphs (Pfennig 1992; Sinervo et al. 2000; Kingsolver et al. 2002; Schluter et al. 2003; Bolnick 2004; Olendorf et al. 2006; Bleay et al. 2007; Pfennig et al. 2007; Weeks and Hoffmann 2008; Mappes et al. 2008; Calsbeek et al. 2009; Kusche et al. 2012; Bolnick and Stutz 2017) and there are still few studies spanning multiple habitats, traits, time periods, and species. In the few studies at these larger scales, rare transgressive hybrid phenotypes appear to suffer a fitness cost, not an advantage (Keagy et al. 2015; Martin 2016a). For example, in a hybrid mesocosm experiment investigating male‐male competition in stickleback, the rarest transgressive phenotypes experienced the lowest reproductive success (Keagy et al. 2015), in contrast to predictions of sexual selection as a diversifying force (Seehausen and Schluter 2004; but see Servedio and Burger 2014; Kopp et al. 2018; Puebla et al. 2012). We previously estimated multiple fitness peaks driving adaptive radiation in the San Salvador Island (SSI) pupfishes by measuring the growth and survival of lab‐reared F2 intercross and backcross hybrids placed in high‐ and low‐density field enclosures (Martin and Wainwright 2013b). Hybrid phenotypes resembling the widespread generalist species were isolated by a local fitness peak, separated by a fitness valley from a higher fitness peak corresponding to hybrid phenotypes resembling the molluscivore specialist, whereas hybrid phenotypes resembling the scale‐eating specialist suffered the lowest growth and survival. Interestingly, rare hybrid phenotypes in this experiment did not experience a survival advantage as predicted by competitive speciation theory. Instead, only hybrids resembling the generalist phenotype experienced a frequency‐dependent survival advantage; rare transgressive hybrids suffered the lowest survival (Martin 2016a). These few experimental studies spanning multiple species suggest that negative frequency‐dependent disruptive selection may primarily operate within a population, rather than among species occupying divergent ecological niches (Martin 2016a; Martin and Richards 2019); however, experimental tests are needed.

Here, we experimentally manipulated the frequency of rare hybrid phenotypes to investigate the static and dynamic features of fitness landscapes. We use a tractable system for empirical measurements of the fitness landscape, an adaptive radiation of pupfishes endemic to San Salvador Island, Bahamas. We first generated transgressive hybrid phenotypes by multiple rounds of backcrossing and intercrossing between a generalist and two trophic specialist *Cyprinodon* pupfish species. We then experimentally manipulated the frequency, but not the overall density, of rare and transgressive hybrid phenotypes between two seminatural field enclosures. This manipulation was performed independently in two ecologically distinct lakes over different exposure periods and seasons using independent hybrid populations originating from each lake. To assess fitness, we individually tagged hybrids, tracked their survival and growth rates in each enclosure, and measured their phenotype from 30 external traits, resulting in estimates of the fitness landscape in four independent field enclosures, two in each lake. This experimental procedure created a normally distributed but highly variable distribution of hybrid phenotypes, recreating the early stages of diversification before adaptive radiation began on San Salvador Island, which originated predominately from Caribbean‐wide standing genetic variation that predates the age of the radiation (McGirr and Martin 2017, 2020; Richards and Martin 2017; Richards et al. 2020). We found negligible effects of the frequency of rare transgressive phenotypes on the survival fitness landscape in both lakes. However, combined inferences across all four field enclosures supported multiple fitness peaks for generalists and molluscivore phenotypes and a large fitness valley isolating the novel scale‐eating specialist, strikingly similar to survival patterns observed in a previous independent field experiment. These results provide strong empirical field support for stasis of major features of a complex fitness landscape, which helps explain the rare evolution of scale‐eating among fishes and the origins of ecological novelty.

## Methods

### FREQUENCY MANIPULATION WITHIN FIELD ENCLOSURES

**Figure 1:**
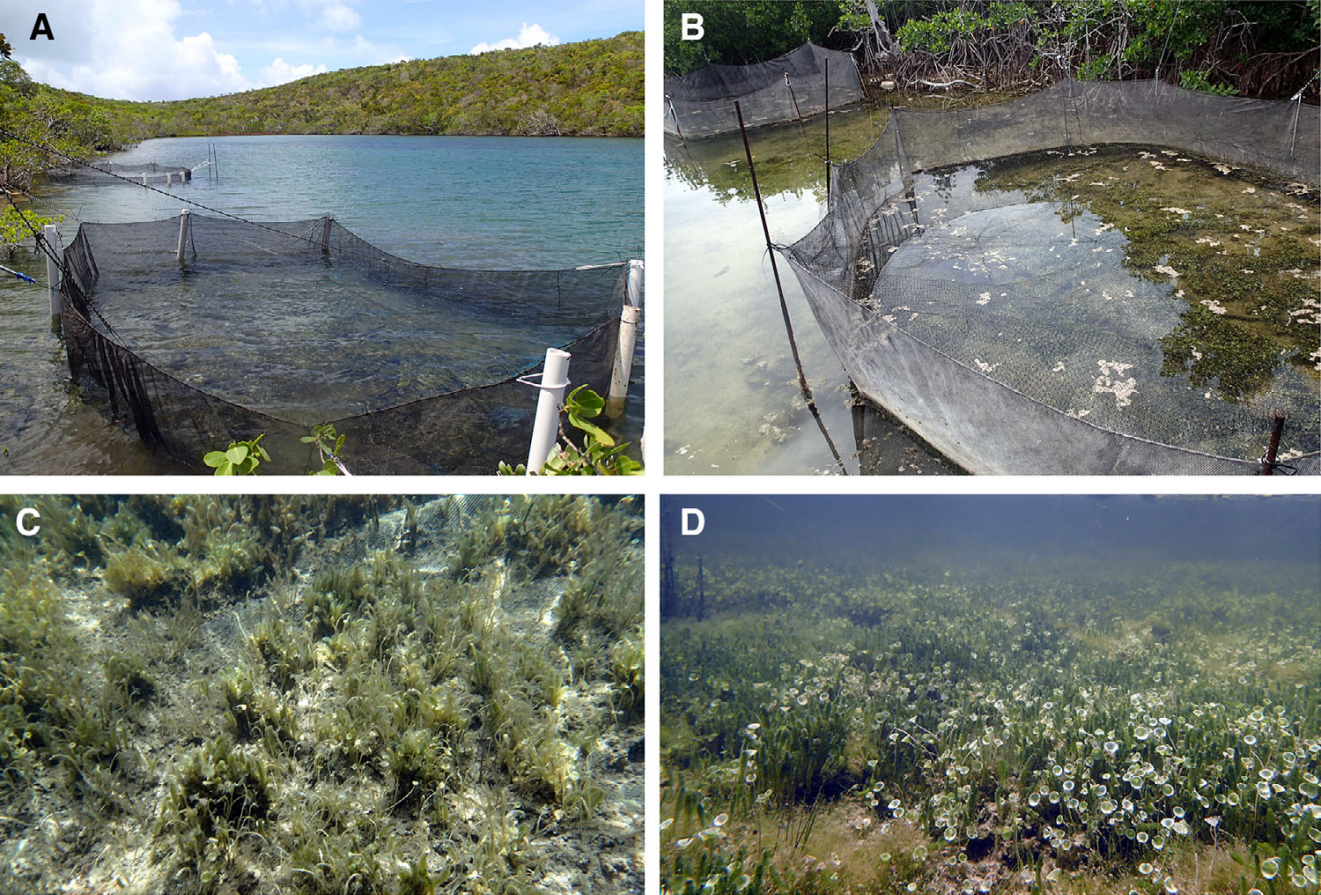
High‐ and low‐frequency field enclosures and the associated benthic macroalgae communities inside each enclosure typical of surrounding littoral zone habitats in lake 1 (A and C: Crescent Pond) and lake 2 (B and D: Little Lake) after 3 month and 11 month field exposure periods, respectively. Each lake contained a high‐ and low‐frequency field enclosure (four total) in which the frequencies of rare transgressive hybrid phenotypes versus common generalist phenotypes were altered by eye during tagging while maintaining comparable overall densities.

We individually tagged and photographed 3407 F4‐F5 outbred juvenile hybrids resulting from crosses of all three species, generalist (*Cyprinodon variegatus*), molluscivore (*Cyprinodon brontotheroides*), and scale‐eater (*Cyprinodon desquamatory*; Martin and Wainwright 2013a), from two different isolated lake populations on San Salvador Island, Bahamas. Tagged hybrids were then released into one of two field enclosures in each lake containing either a high‐ or low‐frequency of rare transgressive hybrid phenotypes (Fig. 1; Table S1). Due to logistical constraints, hybrids could not be measured before the experiment and were sorted by eye into treatments by selectively choosing more transgressive/rare phenotypes for high‐frequency enclosures and more generalist‐like phenotypes for low‐frequency enclosures from a common pool of hybrids from each lake population during the tagging procedure. This was performed in an alternating and haphazard manner over several days (see methods in Supporting Information) so that no treatment group was tagged in a single consecutive block. Our manual frequency manipulation procedure successfully increased the frequency of rare transgressive hybrid phenotypes in the high‐frequency treatments and abundant generalist‐type hybrids in the low‐frequency treatments, respectively, resulting in a significant reduction in phenotypic variance on discriminant axis 2 (predominantly nasal protrusion) in lake 1 (Levene's test, *P* < 0.0001) and discriminant axis 1 (predominantly oral jaw size) in lake 2 (Levene's test, *P* < 0.0001) within the bivariate discriminant morphospace separating all three parental species. This shift in significant discriminant axes between lake populations likely reflects intraspecific differences in genetic architecture, nasal protrusion distance, and gene expression between these lake populations (Martin and Feinstein 2014; McGirr and Martin 2019, 2020). The total density of hybrids was held approximately constant between high‐ and low‐frequency treatments (lake 1: high/low: 923/833 individuals; lake 2: high/low: 842/819 individuals; Table S1; note that only a random subset of 451 and 404 individuals were measured from the total released into lake 2 field enclosures due to low survivorship, which included all survivors plus a random subsample of deaths; see methods in Supporting Information).

### SURVIVAL IN FIELD ENCLOSURES AND LABORATORY CONTROL ENVIRONMENTS

Exposure period was confounded with lake environment in this study, but separating these effects was not our aim. We measured hybrid survival after 3 months in lake 1 (high‐frequency: 77.1% survival; low‐frequency: 75% survival) and after 11 months in lake 2 (high‐frequency: 1.4% survival; low‐frequency: 1.2% survival; Table S1), in the latter case spanning most of the adult pupfish lifespan (Martin et al. 2016) but avoiding mortality due to senescence. There were no differences in survival probability between treatments in each lake (two‐way logistic regression, treatment effect: *P* = 0.237). For a control comparison, additional hybrids from each lake population (*N* = 199 total individuals) were simultaneously tagged, raised in two laboratory aquaria, and their deaths and growth rates were tracked over 1 year concurrently with field experiments.

### MORPHOMETRICS

**Figure 2:**
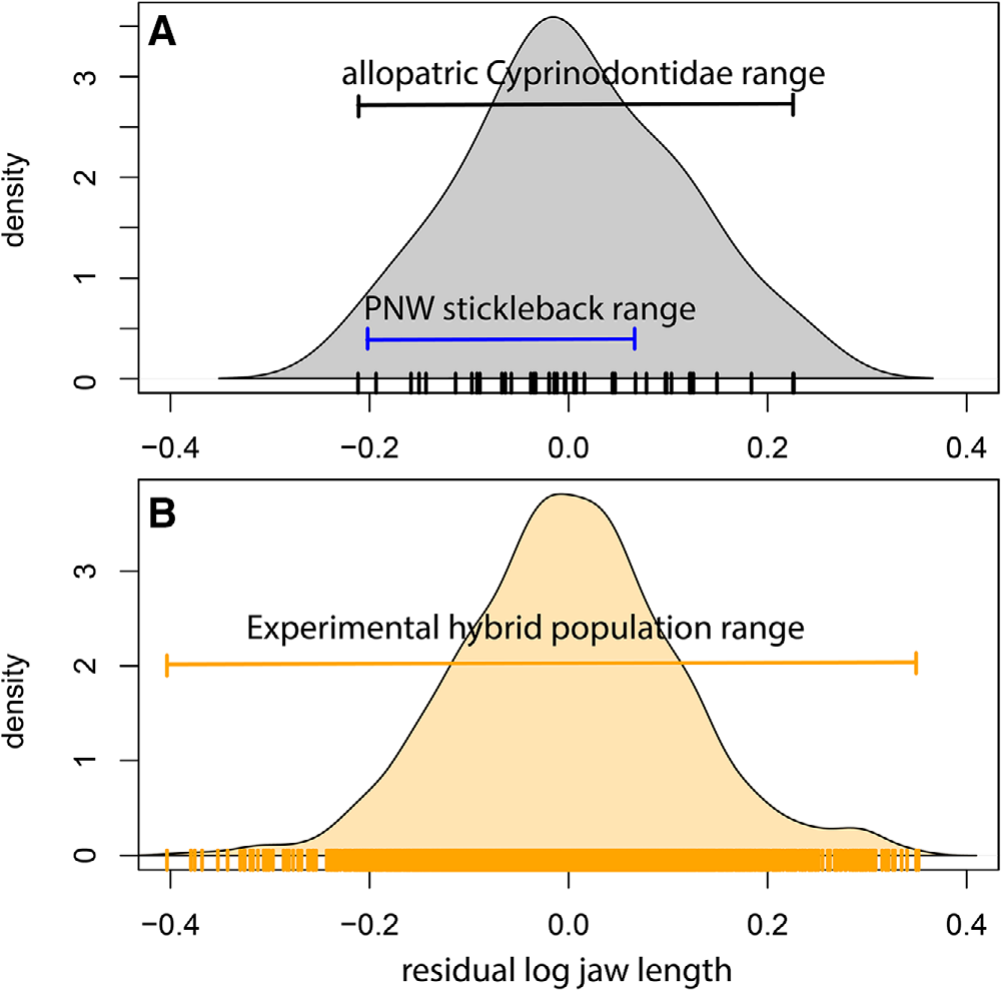
Residual jaw length variation is comparable between (A) allopatric trophic generalist *Cyprinodontidae* species (gray) that diverged 20 million years ago (Furness and Reznick 2015; data from Martin and Wainwright 2011) and (B) lab‐reared F4/F5 hybrid populations (orange) measured from the field experiment in this study. Lab‐reared hybrid variation exceeds the minimum and maximum residual upper jaw lengths across allopatric stickleback populations in the Pacific Northwest (PNW; data from fig. 1 reported in Lavin and McPhail 1986), which have frequently been the focus of previous field experiments supporting negative frequency‐dependent disruptive selection (Schluter et al. 2003; Bolnick 2004; Bolnick and Stutz 2017). Each (A) species mean or (B) individual F4/F5 hybrid is represented by tick marks on the *x*‐axis of the density plots. Residuals were calculated from a single pooled linear regression of log‐transformed lower jaw length on log‐transformed standard length without mean and variance standardization for comparison on the same absolute scale across all three systems.

Phenotypic similarity of each hybrid to the three parental species in each lake (*n* = 236 total lab‐reared parental individuals measured across both lakes; 33 scale‐eaters, 20 molluscivores, and 80 generalists from lake 1; 48 scale‐eaters, 25 molluscivores, and 30 generalists from lake 2) was calculated from 30 linear traits and angles measured from three prerelease photographs of each fish (Fig. S2). These traits were used to estimate two linear discriminant (LD) axes with major loadings of oral jaw size (LD axis 1) and nasal protrusion (LD axis 2; Table S2), diagnostic traits of each specialist species and major axes of trait diversification within this radiation (Martin and Wainwright 2011; Martin 2016b). Indeed, after correcting for standard length, residual jaw length variation within our hybrid populations exceeded the range of variation observed across allopatric *Cyprinodon* species and outgroup Cyprinodontidae species spanning over 20 million years since their most recent common ancestor (data from Martin and Wainwright 2011; Fig. 2).

### SUMMARY OF STATISTICAL ANALYSES

Fitness landscapes were visualized in each enclosure by fitting thin‐plate splines to the survival (binomial) data for each hybrid using generalized cross‐validation, which minimizes residual prediction error of the spline surface. Splines were estimated using the Fields package (Nychka et al. 2017) in R (R Development Core Team 2016). We also used generalized projection pursuit regression to estimate the two major phenotypic axes most strongly associated with survival across the 30‐trait morphospace (Tables S3 and S4; Mitchell‐Olds and Shaw 1987; Schluter and Nychka 1994; Morrissey 2014).

We formally tested for experimental treatment effects on fitness landscapes using generalized additive modeling (GAM) in the mgcv package (Wood 2017). This semiparametric modeling framework enables incorporation of spline terms into generalized linear models and tests of heterogeneity in selection surfaces across treatments, further discussed in Reynolds et al. (2016). We tested the power of this approach to detect frequency‐dependent selection by simulating 1%, 10%, and 20% nonlinear survival increases on one edge of the observed low‐frequency fitness landscapes (Fig. S8) and counting the number of times that a true frequency‐dependent effect was detected in our GAM at an alpha = 0.05 significance threshold over 1000 binomial samples of survivors and deaths from these surfaces. In each sample, we held our observed sample sizes and hybrid phenotype distributions constant.

To examine the local scale of frequency‐dependence within each enclosure, we measured the frequency of similar hybrid phenotypes in local regions of morphospace (Martin 2016a). Within each enclosure, we estimated the Mahalanobis distance for each hybrid individual, the distance to the mean hybrid phenotype in the 30‐dimensional morphospace correcting for trait covariances. We also calculated the sum of the Euclidean distance to the 10 nearest neighbors (i.e., the 10 most similar hybrid phenotypes) in 30‐dimensional morphospace.

It is possible that some regions of the high‐dimensional trait space may still connect scale‐eater phenotypes to other regions of the morphospace through a fitness ridge (Gavrilets 1997, 1999). To further explore the relative fitness of scale‐eater hybrids, we visualized selection across all directions in the 30‐trait morphospace by repeatedly sampling a random subset of 15 traits, calculating a discriminant axis for scale‐eaters relative to generalists within this subspace, and estimating a survival spline for hybrid phenotypes on each arbitrary multivariate axis (Fig. 6). Although we did not measure all traits potentially affecting fitness, this procedure creates a random set of discriminant morphospaces to visualize all fitness pathways across our set of thirty morphological traits. Importantly, this procedure aligns these multivariate linear axes in the same direction from generalist to scale‐eater phenotype enabling comparison of survival curves across random subsets of trait data. Further details on rearing conditions, field enclosures, morphometrics, and statistical analyses are provided in the Supporting Information.

## Results

### STRONG DIRECTIONAL AND NONLINEAR SELECTION FOR GENERALIST AND MOLLUSCIVORE PHENOTYPES

**Figure 3:**
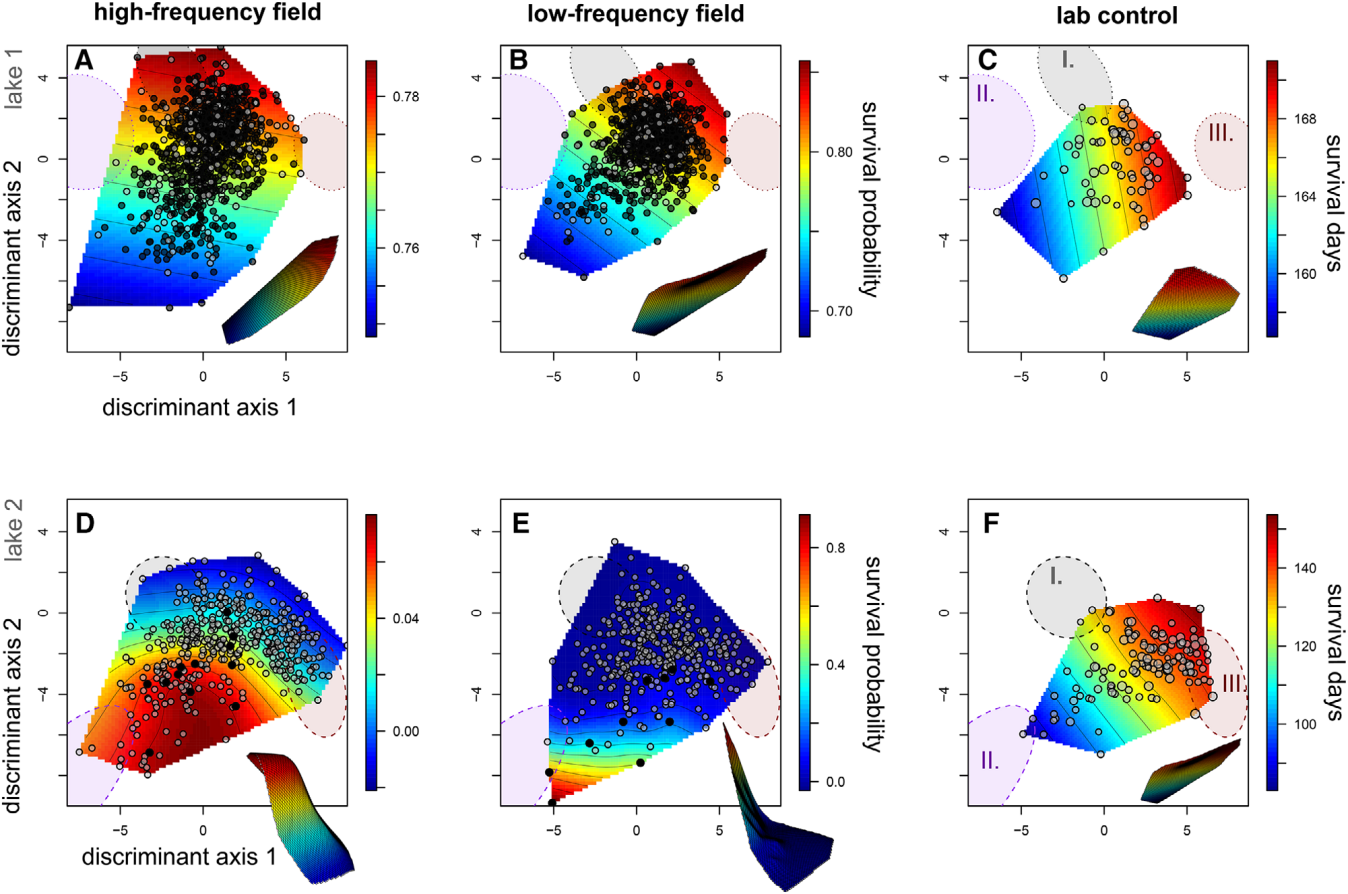
Survival fitness landscapes for hybrid populations in high‐ (first column) and low‐frequency (second column) field enclosures and laboratory controls (third column). Thin‐plate splines predict the probability of survival (heat color) across a single linear discriminant morphospace separating generalist and scale‐eater phenotypes (*x*‐axis: LD1) and generalist and molluscivore phenotypes (*y*‐axis: LD2). Survivors in field enclosures are depicted in black relative to deaths over the 3‐month and 11‐month exposure periods, respectively. Laboratory control points are proportional to the number of days each hybrid survived within 151‐L aquaria. All hybrids are plotted within a shared linear discriminant morphospace for parental species’ phenotypes calculated from lab‐reared F1 individuals of all three parental populations from both lakes (first row: lake 1; second row: lake 2); 95% confidence ellipses for each parental population in each lake are shown for generalists (I. gray), molluscivores (II. purple), and scale‐eaters (III. red); note that molluscivores in lakes 1 and 2 show large differences in mean nasal protrusion distance (Martin and Feinstein 2014) leading to their separation along discriminant axis 2. Survival surfaces are depicted in three dimensions in the offset images.

Selection surfaces for survival in lake 1 were flat and generally favored hybrid phenotypes most similar to generalists in the high‐frequency treatment or generalist and scale‐eater phenotypes in the low‐frequency treatment in lake 1, but this relationship was not significant (binomial GAM with effect of discriminant morphospace spline on survival: *P* > 0.05; Fig. 3). In contrast, we found significant evidence of nonlinear divergent selection for hybrid phenotypes resembling the molluscivore in both high‐frequency (binomial GAM with effect of discriminant morphospace thin‐plate spline on survival: *P* = 0.045) and low‐frequency (*P* = 0.0002) treatments in lake 2, despite lower survival rates over the longer 11 month exposure period in this lake (Fig. 3). Fitness landscapes in lake 2 were estimated from few survivors (*n* = 22); however, random permutation of survival in lake 2 showed that the probability of observing nonlinear selection equal to or more extreme than we observed (GAM *χ* ^2^ ≥ 7.19) due to chance was *P* = 0.014 in the high‐frequency enclosure and *P* < 0.000 (*χ* ^2^ ≥ 21.36) in the low‐frequency enclosure. Patterns of survival in the wild were contrasted by strong directional selection for hybrids resembling the scale‐eater in laboratory control populations from both lakes (poisson GAM with effect of discriminant morphospace thin‐plate spline on days survived in lab aquaria: *P* < 0.00001; Fig. 3).

**Figure 4:**
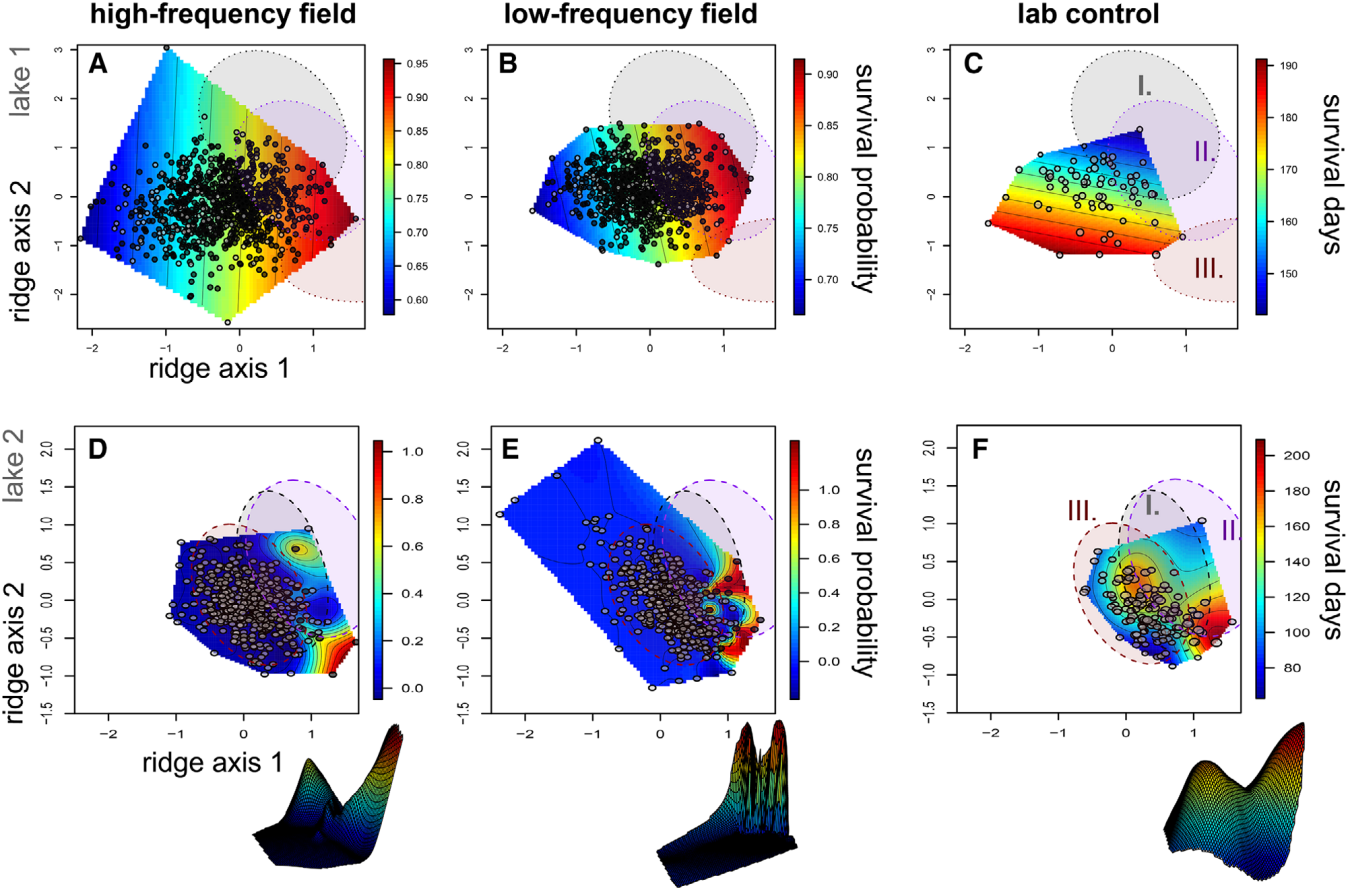
Survival fitness landscapes for hybrid populations in high‐ (first column) and low‐frequency (second column) field enclosures and laboratory controls (third column) across the two major ridge axes of nonlinear selection within the 30‐trait morphospace. Thin‐plate splines predict the probability of survival (heat color) across the two major ridge axes associated with survival (Tables S3 and S4), estimated separately for lake 1 (first row) and lake 2 (second row) hybrid populations using generalized projection pursuit regression (Tables S3 and S4). Survivors in field enclosures are depicted in black relative to deaths over the 3‐month (first row) and 11‐month (second row) exposure periods, respectively (Table S1). Laboratory control points (third column) are proportional to the number of days each hybrid survived within 151‐L aquaria; 95% confidence ellipses for each parental population in each lake are shown for generalists (I. gray), molluscivores (II. purple), and scale‐eaters (III. red). Nonlinear survival surfaces are depicted in three dimensions in the offset images.

We also visualized and analyzed selection on the survival fitness landscapes across all 30 traits in our dataset by estimating the two strongest axes of nonlinear selection using projection pursuit regression. The most transgressive hybrid phenotypes (i.e., least similar to parental phenotypes) suffered the lowest survival probability across treatments in both lakes along these axes (Fig. 4; Table S5: *P* < 0.00001). In contrast, survival in laboratory control populations was shifted away from the direction of selection in field enclosures (Fig. 4). We found significant evidence of directional selection on the strongest nonlinear axis in both lakes (*P* < 0.00001) and marginal evidence of directional selection on the second strongest axis only in lake 2 (Table S5). The traits with the highest loadings on axis 1 were (a) lower jaw length and (b) distance from the jaw joint to the orbit. The highest loadings on axis 2 were (a) angle between the premaxilla and orbit and (b) distance from the premaxilla to the pectoral girdle (Tables S3 and S4), further supporting strong selection on craniofacial trait diversification within this radiation.

### NO EVIDENCE OF FREQUENCY‐DEPENDENT SURVIVAL DIFFERENCES BETWEEN TREATMENTS

We found no evidence that manipulating the frequency of rare transgressive hybrid phenotypes between field enclosures in each lake affected either survival probability or the overall topography of the survival fitness landscapes within the discriminant morphospace (Table S6). Adding a fixed effect for this frequency treatment did not improve the fit to the survival data in any of the generalized additive models examined. Instead, models without the effect of frequency treatment were strongly favored (Table S6; ΔAIC = 11). The power of our GAM modeling framework to detect a significant effect of frequency manipulation on the fitness landscape was 97% for an absolute survival increase of 20% in one region of the low‐frequency field enclosures, 64% for a 10% survival increase, and 16% for a 1% increase in survival (calculated from 1000 binomial sampling draws from simulated fitness surfaces depicted in Fig. S8). Twenty percent survival differences are still relatively minor compared to the 80% survival difference observed in lake 2 low‐frequency enclosure (Fig. 3) and these power simulations were robust among the best generalized additive models compared in Table S6. Thus, given the steep clines in survival observed, we conclude that there was a minimal effect of the frequency manipulation on the survival fitness landscapes measured.

Similarly, we found no evidence for treatment effects on survival along the two major axes of nonlinear selection in either lake estimated from generalized projection pursuit regression (Table S7). Models without the effect of treatment provided a comparable fit to the survival data on the two major axes of nonlinear selection in lake 1 (ΔAIC = 1) and were marginally supported in lake 2 (Table S7; ΔAIC = 1.8).

### LOCAL SCALE OF FREQUENCY DEPENDENCE FOR GROWTH RATE BUT NOT SURVIVAL

Hybrids may only compete with other hybrid phenotypes most similar to their own. The frequency of hybrids with similar phenotypes in neighboring regions of the discriminant morphospace was not significantly associated with residual variation in survival (Fig. S4; Table S6). Generalized additive models including the fixed effect of rare hybrid frequency (distance to mean phenotype) did not meaningfully improve the fit to the survival data (Table S6; ΔAIC = 1; similar results were found for nearest‐neighbor Euclidean distance: Fig. S5).

In contrast, generalized additive models including the fixed effect of rare hybrid frequency (both Mahalanobis and nearest‐neighbor distance) strongly improved the fit to the growth data in lake 1 within the discriminant morphospace (Table S6; Fig. S6; ΔAIC = 12; lake 2 was excluded from all growth rate analyses due to the low number of survivors). Similarly, including the fixed effect of rare hybrid frequency substantially improved the fit to the growth rate data along the two major axes of nonlinear selection in lake 1 (Table S7; ΔAIC = 3.85).

### JOINT INFERENCE OF A COMBINED FITNESS LANDSCAPE SUPPORTS STRIKING SIMILARITY TO A PREVIOUS INDEPENDENT FIELD EXPERIMENT

**Figure 5:**
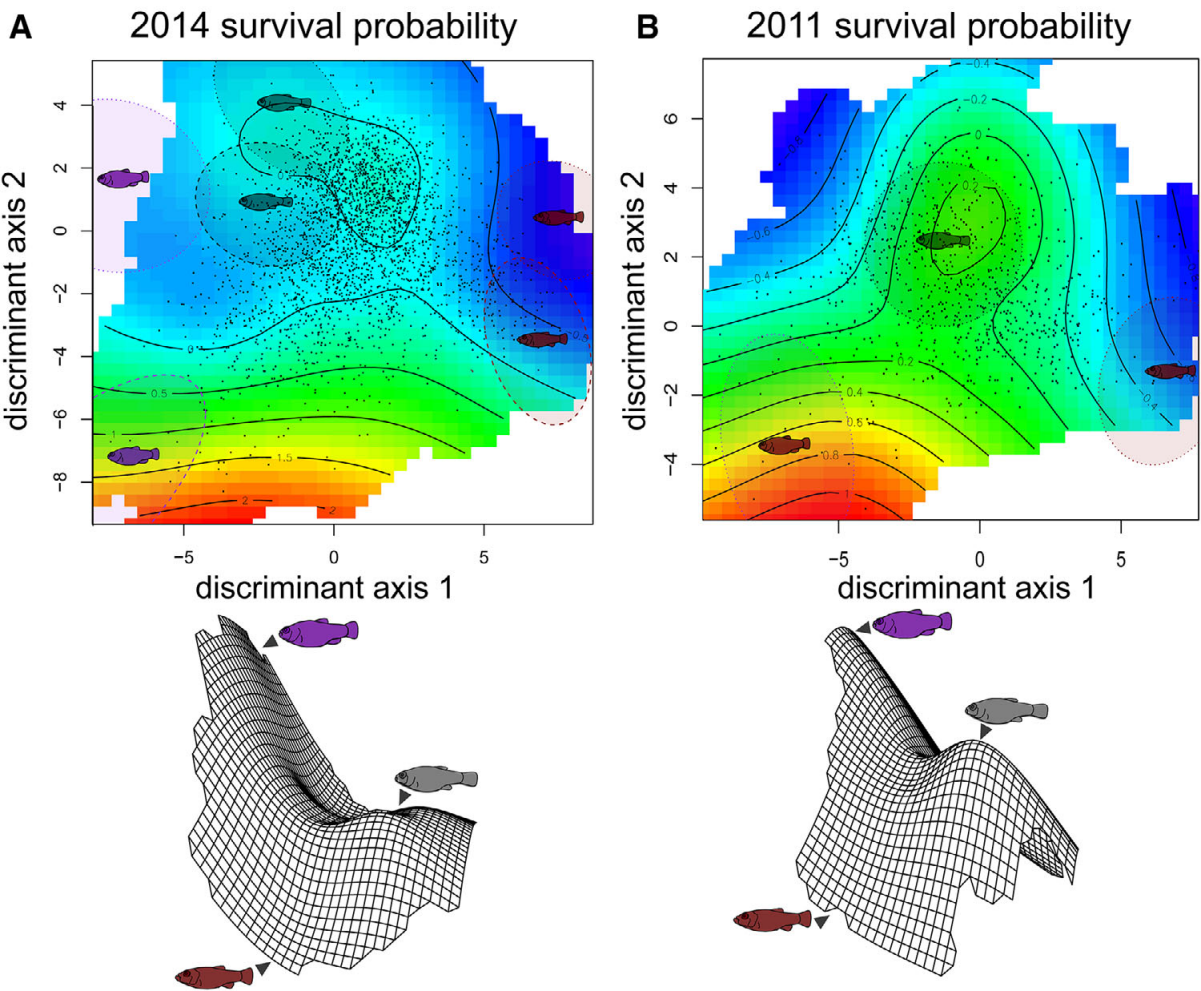
Survival fitness landscapes compared between independent field experiments in (A) 2014 (this study) and (B) 2011 estimated from thin‐plate splines using generalized additive models. Thin‐plate splines in two and three dimensions estimate hybrid survival probability (A) integrating across lake/field exposure period and high‐/low‐frequency treatment for all four enclosures in the 2014–2015 field experiment using a generalized additive model (survival ∼ tps(LD1, LD2) + lake; Table S6); and (B) in only the high‐density field enclosure in lake 1 from the 2011 field experiment (Martin and Wainwright 2013b), calculated from the original data using a generalized additive model (survival ∼ tps(LD1, LD2). The discriminant morphospace separating generalist and scale‐eater phenotypes (*x*‐axis: LD1) and generalist and molluscivore phenotypes (*y*‐axis: LD2) is calculated from lab‐reared individuals of all three parental populations in both lakes in panel A and lake 1 only in panel B; 95% confidence ellipses show the location of generalist (gray), molluscivore (purple), and scale‐eater (red) parental populations from lake 1 (small‐dashed line) and lake 2 (large‐dashed line). Discriminant morphospaces were estimated from independent sets of phenotypic measurements in each panel. The 2014–2015 morphospace was estimated from 30 external traits measured for six parental populations, three from each lake; the 2011 morphospace was estimated independently from a set of 16 external traits (partially overlapping with the 30‐trait set) independently measured for three out of six parental populations (all three lake 1 populations).

GAM enables estimation and visualization of a joint fitness landscape while controlling for the effects of lake/exposure period and frequency treatment in our experiment. Strikingly, the best combined model for survival (across all four treatments in both lakes: Table S2) supported an isolated fitness peak for hybrids resembling the generalist separated by a small fitness valley from a region of higher fitness corresponding to the molluscivore phenotype and a larger region of low fitness corresponding to the scale‐eater phenotype (Fig. 5), similar to a previous independent field experiment in 2011 using F2 hybrids in these same lakes (Martin and Wainwright 2013b). However, in the previous field experiment few of the F2 hybrids fell within the phenotypic range of lab‐reared scale‐eaters (Martin and Wainwright 2013b). In our current experiment, over 70 hybrids occurred within the 95% confidence ellipse of lab‐reared F1 scale‐eater phenotypes in the discriminant morphospace. This provides evidence for environment‐independent features of a complex fitness landscape across years, seasons, divergent lake environments, and experimentally manipulated frequencies of rare transgressive hybrid phenotypes.

Overall, the best supported models for survival included a fixed effect of lake, no treatment effect, and multiple univariate or bivariate thin‐plate splines for the effect of selection (Table S2). Models including spline terms were strongly supported over models including fixed linear effects for the phenotypic discriminant axes (Table S2; ΔAIC = 18.7).

### VISUALIZATION OF HIGH‐DIMENSIONAL FITNESS PATHWAYS

**Figure 6:**
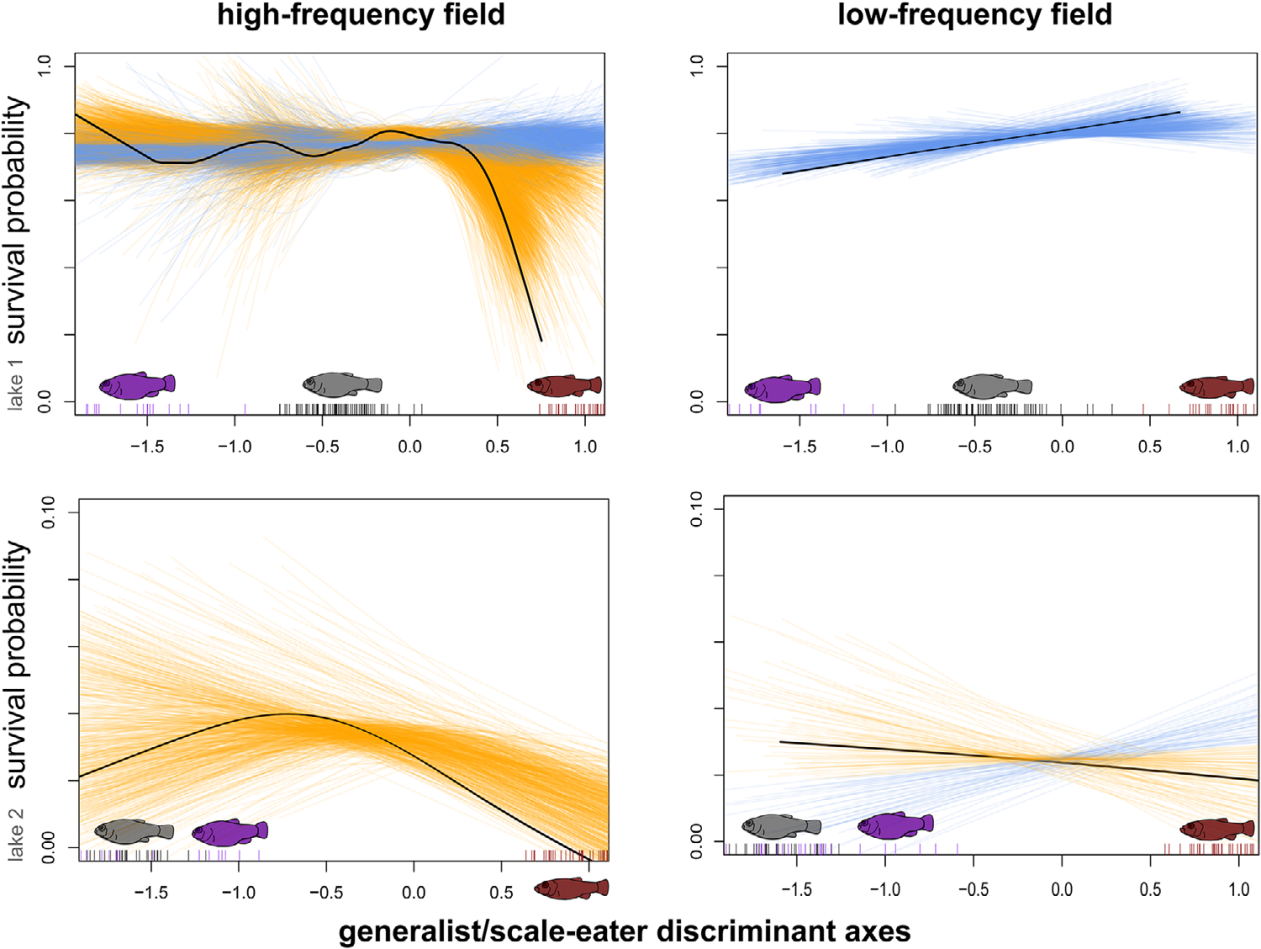
Spaghetti plots illustrate all possible fitness paths between generalist and scale‐eater hybrid phenotypes in high‐ (first column) and low‐frequency (second column) field enclosures. Each orange line depicts the relationship between survival and a random discriminant axis separating generalist and scale‐eater phenotypes estimated from generalized cross‐validation of a smoothing spline for 500 random subsets of 15 size‐corrected traits (out of 30); the black line illustrates the smoothing spline estimated for a discriminant axis from all 30 traits, the orange lines illustrate the smoothing spline estimated for each subset. Each subsampled discriminant axis was rescaled so that the mean parental scale‐eater phenotype = 1 (red arrows). Parental phenotypes are illustrated as black (generalist), purple (molluscivore), and red (scale‐eater) tick marks on the *x*‐axis.

In three out of four field enclosures, hybrids resembling scale‐eaters suffered the lowest survival across the majority of fitness paths subsampled from the 30‐trait morphospace (Fig. 6). This suggests that most fitness pathways, independent of the subsample of traits measured, lead to a fitness minimum isolating the scale‐eater from all other phenotypes. There was no clear signal for dominant trait loadings underlying fitness pathways leading to higher scale‐eater fitness (blue) versus lower scale‐eater fitness (orange) when examining major functional traits such as jaw length, nasal protrusion, adductor height, or head height (Table S8).

## Discussion

### NO EVIDENCE OF RARE PHENOTYPE SURVIVAL ADVANTAGE ON A COMPLEX FITNESS LANDSCAPE

We conducted an experimental field test of frequency‐dependent selection on rare hybrid phenotypes in an adaptive radiation of trophic specialist pupfishes endemic to San Salvador Island, Bahamas. We found no evidence of differences in survival or fitness landscape topography between treatments manipulating the frequency of rare transgressive hybrid phenotypes in two independent lake populations (Figs. 3, 4, and S8; Tables S6–S8; 97% power to detect 20% nonlinear survival differences). We also found no relationship between hybrid survival and the frequency of similar hybrid phenotypes in neighboring regions of morphospace (Figs. S4 and S5; Tables S6 and S7). These patterns were consistent across two important cross‐sections of the 30‐trait morphospace: the two discriminant axes separating the three parental species (Fig. 3; Table S2) and the two major axes of nonlinear selection estimated from generalized projection pursuit regression (Fig. 4; Tables S3‐S4). The lack of any signal of increased survival for rare hybrids suggests that consistent peaks and valleys on the survival fitness landscape are unaffected by the frequency of similar phenotypes and reflect intrinsic viability or organismal performance constraints rather than competitive dynamics. However, it is important to note that growth rate was affected by the frequency of competitors in lake 1 field enclosures (Figs. S4, S6, and S7) and we cannot rule out that additional fitness proxies, such as lifetime reproductive success, may also be affected by competitor frequency. Nonetheless, the stark contrast in frequency dependence between these key life history components, survival and growth, is intriguing and suggests that different selective agents control fitness dynamics and stasis across different life history stages (also see Shaw et al. 2008).

Combined estimates of the survival fitness landscape indicated a surprisingly consistent multipeak topography across two independent field experiments (Fig. 5: 2014 and 2011). Evidence for multiple peaks across lakes resulted from integrating the higher survival of generalist and molluscivore phenotypes after 3 months in lake 1 with the much higher survival of molluscivore phenotypes after 11 months in lake 2 (Fig. 3). Growth rate in lake 1 also showed evidence of multiple peaks due to higher growth rates of generalist phenotypes in both enclosures combined with moderate molluscivore growth rates and low scale‐eater growth rates in the low‐frequency enclosure (Fig. S7). These joint landscapes combine different frequency treatments, exposure periods, lake environments, and potentially different selective regimes. However, we are most interested in estimating the fitness landscape driving adaptive radiation across lake environments, rather than lake‐specific local adaptive regimes unrelated to species divergence. Furthermore, generalized additive models provided no evidence of different selective regimes between treatments or different fitness landscape topography between lakes (Table S6: very low support for models including a lake by thin‐plate spline interaction term), supporting our inference of a single combined selective environment across lakes.

Across all four field enclosures and both fitness proxies, the most consistent feature of fitness landscape topography was a large fitness valley isolating hybrids resembling the scale‐eater. The fitness landscape isolated this novel trophic specialist across spatiotemporal contexts: multiple treatments, lake environments, field exposure periods, and nearly all dimensions of the 30‐trait hybrid morphospace (Figs. 3, 4, 5, 6). This makes sense because this species is the most morphologically, ecologically, behaviorally, and genetically divergent specialist in the radiation (Martin and Wainwright 2013a; Lencer et al. 2016; McGirr and Martin 2018, 2020; Martin et al. 2019; St. John et al. 2019, 2020). This persistent multipeak fitness landscape within SSI's hypersaline lakes provides a striking explanation for the rarity of trophic specialization across the Caribbean because it suggests that generalist populations on neighboring islands are isolated on a local fitness optimum unable to colonize higher neighboring fitness peaks due to stabilizing selection opposing trait diversification (Martin and Wainwright 2013b). This is particularly intriguing given the availability of adaptive alleles for trophic specialization segregating in generalist populations on these neighboring islands (Richards et al. 2020).

### EXTRINSIC AND INTRINSIC FACTORS COULD EXPLAIN LOW SURVIVAL OF TRANSGRESSIVE HYBRID PHENOTYPES

In contrast to predictions of negative frequency‐dependent disruptive selection (Rosenzweig 1978; Gigord et al. 2001; Bolnick 2004; Haller and Hendry 2014), rare transgressive hybrid phenotypes suffered the lowest survival (Figs. 3 and 4). One explanation is that intrinsic viability and organismal performance were impaired due to hybrid ancestry. Hybridization could result in mismatched craniofacial and behavioral traits leading to poor foraging performance (Parnell et al. 2012; Selz et al. 2014; Barreto et al. 2015). For example, oral jaw length variation of trophic specialist pupfish is not controlled by major effect quantitative trait loci (Martin et al. 2017), suggesting that the largest scale‐eater jaws were not fully recovered in any of our F4/F5 hybrids. Second, F1 hybrid scale‐eaters exhibit scale‐biting strike kinematics typical of generalists, suggesting that hybrid scale‐eating performance may be impaired (St. John, Holzman and Martin 2020). Impaired foraging performance of transgressive hybrids in our field enclosures is further supported because scale‐eater hybrids fed only pellet foods exhibited the highest survival rates in lab aquaria (Figs. 3 and 4), demonstrating that the field environment contributed to the low survival of scale‐eater hybrids. This is consistent with other laboratory studies of hybrid feeding performance (Parnell et al. 2008; Arnegard et al. 2014; Matthews and Albertson 2017; Selz and Seehausen 2019).

Intrinsic genetic incompatibilities in hybrids may also be contributing to their low survival rates, particularly if more transgressive hybrid phenotypes are associated with a greater number or more severe genetic incompatibilities (McGirr and Martin 2019, 2020). Genetic incompatibility loci are known to segregate in wild populations (Fishman et al. 2013; reviewed in Cutter 2012; Coughlan and Matute 2020) and hundreds of genetic incompatibility loci have been found in hybrid zones between swordtail fish species (Schumer et al. 2014; Schumer and Brandvain 2016). In support of this hypothesis, F1 hybrids of SSI specialist species misexpressed 9% of genes in larval tissues, much greater than expected given their sympatry and equivalent in magnitude to crosses between generalist pupfish populations separated by 1000 km (McGirr and Martin 2019, 2020). Although the fitness effects on hybrids are unknown, hybrid gene misexpression has been shown to affect hybrid viability and sterility in other systems (Ortiz‐Barrientos et al. 2007; Renaut et al. 2009; Renaut and Bernatchez 2011; Mack et al. 2016; Mack and Nachman 2017) and misexpressed genes in SSI hybrids were enriched in networks affecting craniofacial morphology, muscle mass, and nitrogen metabolism (McGirr and Martin 2020). However, the link between transgressive hybrid phenotypes, divergent ecological selection, and the amount of gene misexpression and misregulation is still unknown (Kulmuni and Westram 2017).

### BIOMECHANICAL AND PHYSIOLOGICAL CONSTRAINTS OF SCALE‐EATING UNDERLIE THE LARGE AND STABLE FITNESS VALLEY ISOLATING THIS ECOLOGICAL NICHE

The rare evolution, high performance demands, and low caloric payoffs of scale‐eating suggest that a wide and deep fitness valley may isolate this niche from all other ecological niches. First, scale‐eating (lepidophagy) is a particularly rare trophic niche among fishes and has evolved independently only 19 times across diverse marine, coastal, riverine, and lacustrine environments (Sazima 1983; Martin and Wainwright 2013c; Kolmann et al. 2018; St John et al. 2019; Grubh and Winemiller 2004; Janovetz 2005; Koblmüller et al. 2007; Raffini and Meyer 2018; Hori 1993). Scale‐eating only evolved once in Cyprinodontiformes and is separated by 168 million years of evolutionary time from the most closely related African cichlid scale‐eating specialists, providing a quantitative measure of the ecological novelty of this niche (Martin and Wainwright 2013c). Second, scale‐eating pupfish perform frequent, rapid strikes resulting in only a few scales and mouthful of skin mucus (St. John and Martin 2019). Thus, the energetic payoff from scale‐eating is likely low relative to performance demands, consistent with the observation that all scale‐eaters are size limited relative to their prey, unlike piscivorous fishes that generally grow much larger than their prey (Sazima et al. 1983).

## Conclusion: On the Origins of Novelty During Adaptive Radiation on Multiple Fitness Peaks

The sensitivity of fitness landscape topography to the environment is rarely measured beyond a single population or fitness peak. Here, we experimentally tested whether manipulating the frequency of rare hybrids phenotypes altered a complex fitness surface driving a diverse radiation of trophic specialist pupfishes, comparable in phenotypic diversity to outgroup species spanning 20 million years. Hybrid survival showed no signal of frequency dependence, in contrast to predictions of competitive speciation theory and previous experiments conducted within a single population or species pair. Furthermore, multiple fitness peaks and a large fitness valley isolating the scale‐eating phenotype were surprisingly stable features shared across different lake environments, competitor frequencies, and two independent field experiments. This challenges our existing view of empirical fitness surfaces as highly sensitive to environmental perturbation (e.g., Grant and Grant 2002). Instead, a multi‐peak fitness landscape spanning macroevolutionary levels of phenotypic disparity displays both dynamic and surprisingly static features across space, time, and competitive environment. These empirical results strengthen the connection between microevolutionary dynamic processes and macroevolutionary stasis by investigating the complex interplay between organismal phenotype and environment within a rapidly diversifying radiation.

## DATA ARCHIVING

All data used for this study will be deposited in the Dryad Digital Repository upon acceptance of the manuscript. https://doi.org/10.5061/dryad.95x69p8hz.

## ETHICS STATEMENT

The Bahamas Environmental Science and Technology Commission and the Ministry of Agriculture kindly provided permission to export, import, and tag fish and conduct this research (research permits renewed annually from 2011 to 2018). All animal care protocols were approved by the University of California, Berkeley and the University of North Carolina at Chapel Hill Animal Care and Use Committees.

## AUTHOR CONTRIBUTIONS

CHM designed the study, performed field experiments, analyzed the data, wrote and edited the manuscript, and funded the study. KG contributed substantially to laboratory data collection.

Associate Editor: Z. Gompert

## Supporting information


**Table S1**. Sample sizes for high‐frequency and low‐frequency field enclosures and laboratory controls for each lake hybrid population.
**Table S2**. Trait loadings on the two linear discriminant axes maximizing phenotypic separation among F1 lab‐reared individuals of the three parental species from both lakes.
**Table S3**. Trait loadings on the two ridge axes most strongly associated with survival probability within the Crescent Pond high and low‐frequency field enclosures estimated using generalized projection pursuit regression.
**Table S4**. Trait loadings on the two ridge axes most strongly associated with survival probability within the Little Lake (lake 2) high and low‐frequency field enclosures estimated using generalized projection pursuit regression.
**Table S5**. Directional selection gradients (β) and matrix of quadratic and correlational selection
gradients (γ) on the two main ridge axes of selection estimated using generalized projection pursuit regression on the 30‐trait morphological dataset for each lake (frequency treatments pooled based on model selection evidence).
**Table S6**. Model selection comparison of general additive models for survival and growth rate of hybrids placed in field enclosures in both lakes.
**Table S7**. Model selection comparison of general additive models for survival and growth rate of hybrids placed in field enclosures in both lakes for the first two major axes of selection (A1 and A2) estimated using generalized projection pursuit projection.
**Table S8**. Proportion of major trait loadings for dominant functional traits along survival fitness pathways leading to lower (orange) or higher (blue) fitness for scale‐eater phenotypes in Fig. 6.
**Figure S1**. Log‐transformed lower jaw length versus log‐transformed standard length for *a)* allopatric Cyprinodontidae species (black) and *b)* hybrid populations (one color per treatment) used in this study.
**Figure S2**. Histograms depicting the phenotypic variance of hybrid populations in high‐ (gray bars) and low‐frequency (orange/blue) treatments in lake 1 (first row) and lake 2 (second row) on the first and second discriminant axes (LD1 and LD2 from Fig. 3).
**Figure S3**. Morphometric landmarks indicating the 28 linear distances, 3 angles (25‐27), and standard length (SL) for *a)* lateral, *b)* close‐up of the craniofacial region, *c)* dorsal view, and *d)* close‐up of the injected coded wire tag in the dorsal musculature including injection site (note different hybrid image used here for clarity).
**Figure S4**. Growth rate fitness landscapes for a) high‐frequency and b) low‐frequency treatments in lake 1 (Crescent Pond).
**Figure S5**. Residual survival probability relative to the density of similar hybrid phenotypes in high‐ (first column) and low‐frequency (second column) field enclosures.
**Figure S6**. Residual growth rate relative to the density of similar hybrid phenotypes in high‐ (first column) and low‐frequency (second column) field enclosures.
**Figure S7**. Joint survival (first column) and growth (second column) fitness landscapes estimated across treatments and lake environments using generalized additive modeling.
**Figure S8**. Simulated 1%, 10%, and 20% effect sizes in the upper region (top fifth) of the survival fitness landscapes for both low‐frequency field enclosures.
