# Supplementary material for: Surprising spatiotemporal stability of a multi‐peak fitness landscape revealed by independent field experiments measuring hybrid fitness
Source: Evol Lett. 2020 Oct 21;4(6):530–44. doi: 10.1002/evl3.195 (PMC7719547; doi:10.1002/evl3.195)
Supplement: Supplementary file 1 — Table S1. Sample sizes for high‐frequency and low‐frequency field enclosures and laboratory controls for each lake hybrid population. Table S2. Trait loadings on the two linear discriminant axes maximizing phenotypic separation among F1 lab‐reared individuals of the three parental species from both lakes. Table S3. Trait loadings on the two ridge axes most strongly associated with survival probability within the Crescent Pond high and low‐frequency field enclosures estimated using generalized projection pursuit regression. Table S4. Trait loadings on the two ridge axes most strongly associated with survival probability within the Little Lake (lake 2) high and low‐frequency field enclosures estimated using generalized projection pursuit regression. Table S5. Directional selection gradients (β) and matrix of quadratic and correlational selection gradients (γ) on the two main ridge axes of selection estimated using generalized projection pursuit regression on the 30‐trait morphological dataset for each lake (frequency treatments pooled based on model selection evidence). Table S6. Model selection comparison of general additive models for survival and growth rate of hybrids placed in field enclosures in both lakes. Table S7. Model selection comparison of general additive models for survival and growth rate of hybrids placed in field enclosures in both lakes for the first two major axes of selection (A1 and A2) estimated using generalized projection pursuit projection. Table S8. Proportion of major trait loadings for dominant functional traits along survival fitness pathways leading to lower (orange) or higher (blue) fitness for scale‐eater phenotypes in Fig. 6. Figure S1. Log‐transformed lower jaw length versus log‐transformed standard length for a) allopatric Cyprinodontidae species (black) and b) hybrid populations (one color per treatment) used in this study. Figure S2. Histograms depicting the phenotypic variance of hybrid populations in high‐ (gray bars) and low‐fr [file EVL3-4-530-s001.docx]

**Supplemental methods**

***Focal lakes***

Laboratory breeding colonies of all three species were collected from two different focal lakes (lake 1: Crescent Pond; lake 2: Little Lake) in 2008, 2013, and 2014 using seine nets and hand nets while snorkeling. Crescent Pond is a 500 m x 60 m x 2 m deep hypersaline (40 ppt) lake containing a 0.1 – 0.3 m layer of fine silt on hard carbonate bedrock and is isolated from surrounding lakes by a small limestone ridge (Hagey & Mylroie 1995; Rothfus 2012). Generalists and molluscivores are morphologically more similar in this lake than others due to a shorter nasal protrusion and exhibit a sister relationship across most of their genome (Martin & Feinstein 2014; Richards & Martin 2017). There is only one other fish species in Crescent Pond, *Gambusia hubbsi*. Little Lake is a 2.3 km x 1 km x 4 m deep hypersaline (40 ppt) lake with a substrate consisting of *Cerithium* spp. snail shells and other molluscs and is connected to the larger interior Great Lake system through a sand bar. The lake contains two fish species *Gambusia hubbsi* and *Atherinomorus stipes* and the amphibious *Kryptolebias marmoratus*. Aquatic flora in both lakes is dominated by thick mats of three macroalgal species (*Acetabularia* *crenulata*, *Batophora oerstedii*, and *Cladophora* sp.) and wigeon grass (*Ruppia maritima*); only Little Lake is surrounded by red mangrove (*Rhizophora mangle*) forests (Martin 2016b).

***Experimental hybrid populations***

Independently for each focal lake, laboratories colonies of all three species were crossed in all directions resulting in both outbred F2 intercrosses and backcrosses (as originally described in (Martin & Wainwright 2013a)). Hybrid populations from each lake were raised for an additional one to two generations in a common laboratory environment before backcrossing to independently sampled colonies of each parental species from each lake (collected in 2013 and 2014), resulting in an outbred population of F4 and F5 intercross and backcross hybrids for each lake, each originating from colonies of 10 – 20 wild-caught individuals from all three species.

Over six weeks, approximately 6,000 F4 and F5 hybrids were bred and raised in recirculating 151-liter aquaria at 5-10 ppt salinity, 25 – 29° C, on a diet of newly hatched brine shrimp, commercial pellet foods, frozen seafood, and dried seaweed. 95% water changes were performed every other day. Before release, juvenile hybrids were size-sorted at least twice using 1/8” mesh bags identical to the field enclosure material resulting in a range of SL from 11.2 – 22.0 mm. This ensured that hybrids were too large to escape field enclosures after their release. There was also no significant relationship between hybrid size at initial release and survival probability. Hybrids were packed in oxygen-permeable shipping bags (Kordon, Inc.) and shipped to San Salvador Island, Bahamas in packing crates (Rubbermaid ActionPacker Storage Box) as checked airline baggage.

***Field enclosure experiments***

Hybrids were temporarily held in concrete holding tanks with flow-through seawater at the Gerace Research Centre after arrival. Each individual was again size-sorted through a 1/8” mesh bag, anesthetized in a solution of buffered MS-222 (Finquel, Inc.), photographed on the left, right, and dorsal sides using a Canon EOS 60D with an EF-S 60 mm f/2.8 USM macro lens mounted on a tripod with external flash, and finally injected subcutaneously in the left dorsal musculature with a 1 mm x 0.1 mm stainless steel sequential coded wire tag using a handheld multi-shot injector (Northwest Marine Technologies, Inc.). A fin-clip from the caudal fin of each fish was removed and stored with the archival tag in 100% ethanol at -20° C. Tagged hybrids were allowed to recover for at least 4 days in flow-through holding tanks at the Gerace Research Centre to fully regrow their caudal fins while fed on a diet of newly hatched brine shrimp and commercial pellet foods.

Field enclosures were constructed from 3.6 m x 4.9 m rectangular fully-enclosed bags with a mesh size of 0.318 cm (Christiansen’s Net Company, Inc.) secured to either PVC pipe set in concrete (lake 1) or iron rebar hammered into the substrate (lake 2; Fig. 1). Two enclosures were deployed in the littoral zone of each lake after removing any debris, then the bottom mesh wall was weighted down with rocks and logs covered in macroalgae from the surrounding area and filled with benthic substrate, macroalgae, and wigeon grass from surrounding areas. Care was taken to avoid introduction of any adult fishes, but smaller pupfish and mosquitofish could still enter the enclosure through the mesh. All hybrids were placed in small mesh bags of enclosure material at least twice before release to ensure that they were too large to escape.

One enclosure in each lake was randomly selected as the high frequency treatment and the second enclosure was the low frequency treatment. Independently in each of the two hybrid populations, hybrids were individually selected for each treatment by eye, selecting more divergent transgressive phenotypes for the high-frequency treatment and selecting the most generalist-like hybrids for the low-frequency treatment. This resulted in reduced phenotypic variance and morphospace occupation within the low-frequency treatment within each lake. This also effectively reduced the frequency of scale-eater hybrids falling within the 95% confidence interval of parental scale-eater phenotypes. Densities of the most transgressive hybrids within enclosures fully recapitulating the parental scale-eater and molluscivore phenotypes approximated the natural densities of 0.9% and 3% scale-eaters and 6 and 5% molluscivores in Crescent Pond and Little Lake, respectively (Martin & Wainwright 2013b).

In Crescent Pond (lake 1), tagged hybrids were released in large batches into the high-frequency enclosure on May 15^th,^ 19^th^, and 28^th^ and low-frequency enclosure on May 21^st^, 26^th^, and 28^th^, 2014, respectively. In Little Lake (lake 2), all tagged hybrids were released into the high-frequency enclosure on May 18^th^ and into the low-frequency enclosure on May 25^th^ and 27^th^, 2014. Surviving hybrids were recovered from lake 1 on August 26^th^ and 27^th^, 2014 after 3 months by carefully removing the substrate and sequentially lifting the entire mesh bottom, then photographed laterally and stored in 100% ethanol. To sample from a wider range of seasonal environments and recover the full time to reproductive maturity, surviving hybrids were recovered from lake 2 on April 28^th^ and 29^th^, 2015 after 11 months in field enclosures. Tags were dissected from all survivors after preservation, read using a 100x tag-reading scope from Northwest Marine Technologies, Inc, and matched with archival tags to identify the survival status (0 or 1) of each tagged hybrid.

***Laboratory control***

Additional hybrids from each population (*n* = 199, Table S1) were raised in two 151-liter laboratory aquaria (1 per lake population) concurrently with the field experiment for 11 months. Although these laboratory environments differed in salinity, temperature, and density, hybrids were raised on a control diet of only commercial pellet foods to provide a uniform control dietary resource offering no advantages for specialized trophic morphology. Hybrids were fed once daily an amount of food that could be consumed in five minutes but not *ad libitum* and raised at 26 – 27° C year-round in 5-10 ppt salinity (Instant Ocean) with weekly 95% water changes. Laboratory hybrids grew faster than fish placed in field enclosures at these higher year-round temperatures and high densities within each aquarium population led to strong competition for food and high mortality rates, whereas survivors collected from field enclosures never reached maximum adult sizes or approached senescence. The day of each laboratory death was recorded, followed by removal of the tag to identify the pre-release photograph of that individual. Laboratory deaths were tracked from July 1^st^, 2014 until May 21^st^, 2015.

***Morphometrics***

Each hybrid used in field experimental (*n* = 2,611) and laboratory control populations (*n* = 199) was measured for 21 landmarks (Fig. S3) on both left and right lateral sides and 10 landmarks on the dorsal surface of the head plus a 4 mm size-standard grid for calibration of each image using TpsDig2 (Rohlf 2001). F1 lab-reared individuals of each parental species from each lake (*n* = 236) were also measured in the same way. 27 linear distances and three angles were calculated from these landmarks and then averaged for both lateral sides, resulting in 30 trait measurements plus standard length. Traits were selected to capture phenotypic divergence in craniofacial morphology and body shape (Fig. S3). All three San Salvador Island species are highly divergent in morphology at this late juvenile developmental stage (Lencer et al. 2017).

Linear trait measurements and angles were then imported into R (R Development Core Team 2018) and size-corrected by taking the residuals from a standard major axis regression relative to log-transformed standard length (SL) for each trait in the entire hybrid pool and F1 lab-reared parental individuals from both lakes (n = 110 generalists, 45 molluscivores, 81 scale-eaters) using the sma function in the smatr package (Warton *et al.* 2012) in R. Standard major axis regression is appropriate when trait measurement error is present on the x-axis and the y-axis and is equivalent to the first principal component of phenotypic variance between these two axes (Warton *et al.* 2012). Initial inspection of size-correction plots indicated that ordinary least squares (OLS) regression tended to overestimate the slope of the regression line, particularly for highly variable traits among parental and hybrid populations such as oral jaw length. However, our results were robust to OLS size-correction. No allometric scaling was observed among different species except for nasal protrusion distance and nasal protrusion angle, which exhibited no association with log-transformed SL and were not size-corrected. All size-corrected trait residuals and uncorrected nasal protrusion distance and angle were standardized to a standard deviation of one and mean of zero for comparisons across traits. There was no effect of standard length at introduction on survival of hybrids (GLM logistic regression with effects of field enclosure and log-transformed SL: *P* = 0.709)

***Visualization of fitness landscapes***

Fitness landscapes were visualized in each enclosure by fitting thin-plate splines to the survival (binomial) or growth rate (normal) data for each hybrid using generalized cross-validation (GCV), which minimizes residual prediction error of the spline surface. Splines were estimated using the Fields package (Nychka *et al.* 2017) in R. When over-fitting was apparent, restricted estimation of maximum likelihood (REML) was used to estimate the curvature of the spline instead of GCV (used for both survival landscapes in lake 2; REML estimation of splines was identical to GCV surfaces in lake 1). Growth rate was only examined in lake 1 due to the low number of survivors with growth rate data from lake 2 (Table S1).

We focused on two different cross-sections of the 30-dimensional hybrid morphospace. First, we examined selection on the two-dimensional linear discriminant morphospace maximizing phenotypic separation among the three parental species from both lakes using the lda function in the MASS package in R (Venables & Ripley 2002). This morphospace provides a simple index of hybrid similarity to each of the three parental species, comparable to multivariate hybrid indices from similar studies of hybrid fitness between two species (e.g. (Schemske & Bradshaw 1999; Schluter 2003)). Second, we examined the two major axes of nonlinear selection within the 30-dimensional morphospace using generalized projection pursuit regression for binomial data using the gppr function in the gsg package in R (Morrissey & Sakrejda 2013; Morrissey 2014), following the original recommendations of (Schluter & Nychka 1994). This approach avoids the problematic quadratic assumptions of canonical rotation (Phillips & Arnold 1989; Blows & Brooks 2003), particularly for highly nonlinear relationships between morphology and survival such as in our dataset, and enables visualization of the major axes of nonlinear selection within the dataset. We calculated the first two ridge axes using the gppr function with a binomial family of response distributions and then projected hybrid phenotypes onto each ridge axis by matrix multiplication. We performed generalized projection pursuit regression separately for lake 1 and lake 2 hybrid populations due to the large differences in survival (Table S1).

***Generalized additive modeling***

We formally tested for experimental treatment effects on fitness landscapes using generalized additive modeling in the mgcv package (Wood 2017) in R. This semi-parametric modeling framework enables incorporation of spline terms into generalized linear models and comparisons of models containing spline, fixed, and random effect terms using AIC. This allows for tests of heterogeneity in selection surfaces across treatments, further discussed in Reynolds et al. (2016). For the survival data, we compared models with the fixed effects of treatment and lake and all combinations of univariate smoothing splines and thin-plate splines on both discriminant axes (Table 2) or both major ridge axes of selection estimated from generalized projection pursuit regression (Table 3). We also explored models allowing the thin-plate spline surface (i.e. the fitness landscape) to vary between lake environments using the ‘by’ term within the thin-plate function. Finally, we included models with covariates including log-transformed standard length and distance measures of competitor frequency within each enclosure based on either Mahalanobis distance or nearest-neighbor Euclidean distance (see below). Models were compared using AIC. We examined a similar range of models for the growth rate data, calculated from the difference in log-transformed SL between the pre-release and post-capture photograph of each surviving fish. We excluded lake 2 from all growth rate analyses due to the low number of survivors in this lake.

***Analyses of frequency-dependent selection within enclosures***

We used two approaches to measure the frequency of competitors (i.e. number of hybrids with similar phenotypes relative to a focal hybrid individual) within each enclosure. First, we calculated the Mahalanobis distance from each hybrid phenotype to the mean hybrid phenotype in the full 30-trait morphospace using the mahalanobis function in R. This distance estimates the disparity of each hybrid relative to the most abundant hybrid phenotypes while accounting for trait correlations. Second, we measured the frequency of competitors in the local region of morphospace surrounding each hybrid by calculating the sum of the Euclidean distance to the ten nearest neighbors in the full 30-trait morphospace, following the approach in (Martin 2016a). We used the knn.dist function in the FNN package (Beygelzimer *et al.* 2019) to the calculate the matrix of distances among all hybrid pairs.

***Analyses of a fitness valley for scale-eater phenotypes***

To evaluate the stability of a survival fitness valley near scale-eaters across all dimensions in our 30-trait morphospace, we estimated and visualized smoothing splines for survival from random subsets of the trait dataset. We first estimated a smoothing spline for survival relative to the discriminant axis (LD1 in Fig. 3) for all 30 traits separating parental scale-eater phenotypes from generalist phenotypes. We then randomly drew 15 traits from the dataset and recalculated the generalist-scale-eater discriminant axis and a new survival spline for each of 500 trait subsets using a custom script in R. This enabled alignment of each discriminant vector from generalist to scale-eater regardless of the subset of traits sampled. Each of the 500 discriminant vectors was then rescaled to the mean parental scale-eater phenotype, so that each hybrid could be scored on the same scale proportional to their phenotypic similarity to the scale-eater (i.e. 1 = full match). Survival splines were estimated separately for each field enclosure and plotted on a shared, rescaled generalist-scale-eater discriminant axis to visualize the overall evidence for a fitness valley in the scale-eater region. Splines above or below a fitness threshold within the region of the scale-eater 95% confidence ellipse were colored blue or orange (Fig. 7) and major trait loadings on discriminant axes in these two regions of the fitness hypervolume were summed to assess how trait loadings affected fitness pathways.

**References**

Beygelzimer, A., Kakadet, S., Langford, J., Arya, S., Mount, D. & Li, S. (2019). FNN: Fast Nearest Neighbor Search Algorithms and Applications.

Blows, M.W. & Brooks, R. (2003). Measuring Nonlinear Selection, 162.

Hagey, F. & Mylroie, J. (1995). Pleistocene lake and lagoon deposits, San Salvador Island, Bahamas. *Geol. Soc. Am. Spec. Pap.*, 1995, 77–90.

Lavin, A. & McPhail, J. (1986). Adaptive Divergence of Trophic Phenotype among Freshwater Populations of the Threespine Stickleback (Gasterosteus aculeatus). *Can. J. Fish. Aquat. Sci.*, 43, 2455–2463.

Martin, C.H. (2016a). Context dependence in complex adaptive landscapes : frequency and trait-dependent selection surfaces within an adaptive radiation of Caribbean pupfishes. *Evolution (N. Y).*, 1–18.

Martin, C.H. (2016b). The cryptic origins of evolutionary novelty: 1000‐fold faster trophic diversification rates without increased ecological opportunity or hybrid swarm. *Evolution (N. Y).*, 70.11, 2504–2519.

Martin, C.H. & Feinstein, L.C. (2014). Novel trophic niches drive variable progress towards ecological speciation within an adaptive radiation of pupfishes. *Mol. Ecol.*, 23, 1846–62.

Martin, C.H. & Wainwright, P.C. (2011). Trophic novelty is linked to exceptional rates of morphological diversification in two adaptive radiations of *Cyprinodon* pupfishes. *Evolution*, 65, 2197–212.

Martin, C.H. & Wainwright, P.C. (2013a). Multiple fitness peaks on the adaptive landscape drive adaptive radiation in the wild. *Science*, 339, 208–211.

Martin, C.H. & Wainwright, P.C. (2013b). On the measurement of ecological novelty: scale-eating pupfish are separated by 168 my from other scale-eating fishes. *PLoS One*, 8, e71164.

Mitchell-Olds, T. & Shaw. (1987). Regression analysis of natural selection: statistical inference and biological interpretation. *Evolution (N. Y).*, 41, 1149–1161.

Morrissey, M. & Sakrejda, K. (2013). gsg: an R package for inference of selection gradients.

Morrissey, M.B. (2014). In search of the best methods for multivariate selection analysis. *Methods Ecol. Evol.*, n/a-n/a.

Nychka, D., Furrer, R., Paige, J. & Sain, S. (2017). fields: Tools for spatial data. *R Packag. version 9.6*.

Phillips, P.C.P.C. & Arnold, S.J.S.J. (1989). Visualizing multivariate selection. *Evolution (N. Y).*, 43, 1209–1222.

R Development Core Team. (2016). R: A Language and Environment for Statistical Computing. *R Found. Stat. Comput. Vienna Austria*, 0, {ISBN} 3-900051-07-0.

Richards, E.J. & Martin, C.H. (2017). Adaptive introgression from distant Caribbean islands contributed to the diversification of a microendemic adaptive radiation of trophic specialist pupfishes. *PLoS Genet.*, 13, 1–35.

Rohlf, F.J. (2001). Comparative methods for the analysis of continuous variables: geometric interpretations. *Evolution*, 55, 2143–60.

Rothfus, E. (2012). Water-quality monitoring of San Salvadorian inland lakes. In: *Proceedings of the 15th Symposium on the Geology of the Bahamas and other Carbonate Regions* (eds. Gamble, D. & Kindler, P.). Gerace Research Center, pp. 129–138.

Schemske, D.W. & Bradshaw, H.D. (1999). Pollinator preference and the evolution of floral traits in monkeyflowers (*Mimulus*). *Proc. Natl. Acad. Sci. U. S. A.*, 96, 11910–5.

Schluter, D. (2003). Frequency dependent natural selection during character displacement in sticklebacks. *Evolution*, 57, 1142–50.

Schluter, D. & Nychka, D. (1994). Exploring fitness surfaces. *Am. Nat.*, 143, 597–616.

Stinchcombe, J.R., Agrawal, A.F., Hohenlohe, P. a, Arnold, S.J. & Blows, M.W. (2008). Estimating nonlinear selection gradients using quadratic regression coefficients: double or nothing? *Evolution*, 62, 2435–40.

Venables, W. & Ripley, B. (2002). *Modern Applied Statistics with S*. Fourth Edi. Springer, New York.

Warton, D., Duursma, R., Remko, A., Falster, D. & S, T. (2012). smatr 3 - an R package for estimation and inference about allometric lines. *Methods Ecol. Evol.*, 3, 257–259.

Wood, S. (2017). *Generalized Additive Models: An Introduction with R (2nd edition)*. Chapman and Hall.

**Table S1.** Sample sizes for high-frequency and low-frequency field enclosures and laboratory controls for each lake hybrid population.

| **lake** | **exposure period** | **high-frequency treatment survivors / total** | **low-frequency treatment survivors / total** | **control** |
| --- | --- | --- | --- | --- |
| Crescent Pond | 3 months | 712 / 923; 77.1% | 662 / 883; 75.0% | 69 |
| Little Lake | 11 months | 12 / 842; 1.4% | 10 / 819; 1.2% | 130 |

**Table S2.** Trait loadings on the two linear discriminant axes maximizing phenotypic separation among F1 lab-reared individuals of the three parental species from both lakes. Numbered landmarks correspond to illustrations of linear distances and angles in Fig. S3.

| **trait** | **trait** | **LD1** | **LD2** |
| --- | --- | --- | --- |
|  | cranialwidth | -0.10 | 0.00 |
|  | innereyetosnout | -0.33 | 0.28 |
|  | suspensorium | 0.74 | 0.37 |
|  | dorsalsnoutlen | 0.11 | -0.37 |
|  | adductorht | 0.22 | -0.23 |
|  | jawlen | 1.07 | -0.19 |
|  | ad2pect | -0.07 | 0.16 |
|  | pmxlen | 0.52 | -0.38 |
|  | foreeyewidth | 0.17 | -0.42 |
|  | bodydepth | 0.10 | -0.27 |
|  | dorsaltocaudal | -0.12 | 0.01 |
|  | headht | 0.55 | -0.53 |
|  | analtocaudal | -0.29 | -0.07 |
|  | caudalpedht | -0.19 | 0.29 |
|  | pmx2add | -0.76 | 0.49 |
|  | jaw2pect | -0.10 | -0.07 |
|  | snoutlen | 0.69 | -0.19 |
|  | foresnout | -0.64 | 0.26 |
|  | eyewidth | 0.01 | 0.04 |
|  | eyetosnout | 0.29 | 0.71 |
|  | headwidth | -0.11 | 0.23 |
|  | nose | -1.06 | -1.61 |
|  | hindeyewidth | -0.12 | 0.03 |
|  | eyeht | -0.76 | -0.11 |
|  | topeyeangle | 0.35 | 0.43 |
|  | lowereyeangle | -0.23 | 0.68 |
|  | nasalangle | 0.49 | 0.01 |
|  | headlen | 0.02 | -1.07 |
|  | bellylen | -0.44 | 0.3 |
|  | pectinsertion | -0.19 | -0.19 |
|  | buccalwidth | 0.16 | 0.3 |

**Table S3.** Trait loadings on the two ridge axes most strongly associated with survival probability within the Crescent Pond high and low-frequency field enclosures estimated using generalized projection pursuit regression. Numbered landmarks correspond to illustrations of linear distances and angles in Fig. S3.

| **trait** | **trait** | **ridge axis 1 (A1)** | **ridge axis 2 (A2)** |
| --- | --- | --- | --- |
| 1. nasal protrusion | 1. nose | -0.02 | 0.06 |
| 2. interorbital width | 2. cranialwidth | 0.2 | 0.02 |
| 3. orbit to premaxilla | 3. innereyetosnout | 0.27 | 0.22 |
| 4. suspensorium length | 4. suspensorium | -0.24 | 0.23 |
| 5. dorsal facial length | 5. dorsalsnoutlen | -0.26 | -0.22 |
| 6. adductor height | 6. adductorht | 0.26 | -0.03 |
| 7. lower jaw length | 7. jawlen | 0.21 | 0.42 |
| 8. subopercle to pectoral girdle | 8. ad2pect | -0.11 | -0.26 |
| 9. premaxilla length | pmxlen | -0.25 | -0.31 |
| 10. jaw joint to orbit | foreeyewidth | 0.36 | 0.04 |
| 11. body depth | bodydepth | 0.15 | 0.09 |
| 12. dorsal to caudal distance | dorsaltocaudal | -0.18 | 0.01 |
| 13. head height | headht | 0.17 | -0.35 |
| 14. anal to caudal distance | analtocaudal | -0.02 | 0.1 |
| 15. caudal peduncle height | caudalpedht | -0.11 | 0.17 |
| 16. lateral skull length | pmx2add | -0.13 | -0.17 |
| 17. upper jaw to pectoral girdle | jaw2pect | -0.05 | 0.15 |
| 18. lateral facial length | snoutlen | 0.05 | -0.2 |
| 19. nasal length | foresnout | -0.02 | -0.06 |
| 20. horizontal orbit diameter | eyewidth | -0.07 | -0.08 |
| 21. adductor to premaxilla | eyetosnout | -0.09 | 0.14 |
| 22. max. neurocranium width | headwidth | 0.05 | 0.13 |
| 23. orbital neurocranium width | hindeyewidth | -0.12 | -0.02 |
| 24. vertical orbit diameter | eyeht | 0.13 | 0.23 |
| 25. premaxilla to orbit angle | topeyeangle | -0.23 | 0.14 |
| 26. premaxilla to adductor angle | lowereyeangle | -0.16 | 0.24 |
| 27. nasal protrusion anagle | nasalangle | 0.06 | 0.18 |
| 28. neurocranium to premaxilla | headlen | -0.2 | -0.15 |
| 29. orbit to anal fin insertion | bellylen | -0.22 | 0.03 |
| 30. pectoral fin insertion width | pectinsertion | -0.25 | -0.04 |
| 31. gape width | buccalwidth | -0.23 | -0.12 |

**Table S4.** Trait loadings on the two ridge axes most strongly associated with survival probability within the Little Lake (lake 2) high and low-frequency field enclosures estimated using generalized projection pursuit regression. Numbered landmarks correspond to illustrations of linear distances and angles in Fig. S3.

| **trait** | **trait** | **ridge axis 1 (A1)** | **ridge axis 2 (A2)** |
| --- | --- | --- | --- |
| 1. nasal protrusion | nose | 0.18 | -0.02 |
| 2. interorbital width | cranialwidth | -0.07 | 0.14 |
| 3. orbit to premaxilla | innereyetosnout | -0.08 | 0.24 |
| 4. suspensorium length | suspensorium | -0.19 | 0.12 |
| 5. dorsal facial length | dorsalsnoutlen | 0.2 | -0.33 |
| 6. adductor height | adductorht | 0 | 0.01 |
| 7. lower mandible length | jawlen | -0.26 | 0.11 |
| 8. subopercle to pectoral girdle | ad2pect | -0.13 | 0.02 |
| 9. premaxilla length | pmxlen | 0.13 | -0.04 |
| 10. jaw joint to orbit | foreeyewidth | -0.05 | 0.19 |
| 11. body depth | bodydepth | -0.03 | 0.15 |
| 12. dorsal to caudal distance | dorsaltocaudal | 0.03 | -0.17 |
| 13. head height | headht | 0.04 | -0.07 |
| 14. anal to caudal distance | analtocaudal | -0.07 | -0.18 |
| 15. caudal peduncle height | caudalpedht | -0.05 | -0.15 |
| 16. lateral skull length | pmx2add | -0.23 | 0.22 |
| 17. upper jaw to pectoral girdle | jaw2pect | 0.34 | -0.43 |
| 18. lateral facial length | snoutlen | -0.17 | 0.19 |
| 19. nasal length | foresnout | 0.03 | -0.05 |
| 20. horizontal orbit diameter | eyewidth | 0 | 0.08 |
| 21. adductor to premaxilla | eyetosnout | -0.23 | 0.22 |
| 22. max. neurocranium width | headwidth | 0.08 | -0.08 |
| 23. orbital neurocranium width | hindeyewidth | 0.15 | -0.12 |
| 24. vertical orbit diameter | eyeht | 0.25 | -0.19 |
| 25. premaxilla to orbit angle | topeyeangle | -0.51 | 0.47 |
| 26. premaxilla to adductor angle | lowereyeangle | -0.26 | 0.13 |
| 27. nasal protrusion anagle | nasalangle | 0.02 | 0.01 |
| 28. neurocranium to premaxilla | headlen | 0.21 | 0.02 |
| 29. orbit to anal fin insertion | bellylen | 0.03 | -0.12 |
| 30. pectoral fin insertion width | pectinsertion | 0.12 | 0.04 |
| 31. gape width | buccalwidth | -0.19 | 0.01 |

Table S5. Directional selection gradients (β) and matrix of quadratic and correlational selection
gradients (γ) on the two main ridge axes of selection estimated using generalized projection pursuit regression on the 30-trait morphological dataset for each lake (frequency treatments pooled based on model selection evidence). β and γ were estimated in separate regressions. Directional selection gradients in bold were significant in a one-way logistic regression model with fixed effects of ridge terms. Quadratic coefficients from the multiple regression model were doubled to estimate quadratic selection gradients (Stinchcombe et al. 2008). However, please note that a quadratic approximation is inappropriate to model these highly nonlinear survival data (Mitchell-Olds & Shaw 1987).

| Lake 1 (Crescent Pond) |  |  | **γ** | |
| --- | --- | --- | --- | --- |
| **ppr ridge axis** | **β** | ***N* = 1756** | **A1** | **A2** |
| A1 | 0.095***** |  | -0.050 |  |
| A2 | 0.005 |  | -0.026 | 0.030 |
| Lake 2 (Little Lake) |  |  | **γ** | |
| **ppr ridge axis** | **β** | ***N* = 855** | **A1** | **A2** |
| A1 | 0.126***** |  | .408 |  |
| A2 | 0.052* |  | .185 | .081 |

*P < 0.05, **P < 0.01, ***P < 0.001, *****P < 0.00001

**Table S6.** Model selection comparison of general additive models for survival and growth rate of hybrids placed in field enclosures in both lakes. The best supported model is indicated first. Notation is adopted from the mgcv package in R: s(LD1) indicates a smoothing spline fit to discriminant axis one; tps (LD1, LD2) indicates a thin-plate spline fit to the two discriminant axes; competitor distance indicates the Mahalanobis distance from each hybrid phenotype to the mean phenotype within the 30-trait morphospace while accounting for trait correlations. Growth rate models were only analyzed for lake 1 due to the low survival rates in lake 2.

| **model** | **AIC** | **ΔAIC** |
| --- | --- | --- |
| survival ~ s(LD1) + s(LD2) + lake | 2033.095 | - |
| survival ~ tps(LD1, LD2) + s(LD1) + s(LD2) + lake | 2033.096 | 0.001 |
| survival ~ tps(LD1, LD2) + lake | 2042.247 | 9 |
| survival ~ tps(LD1, LD2) + lake + competitor distance | 2043.28 | 10 |
| survival ~ tps(LD1, LD2) + lake + **treatment** + logSL | 2044.308 | 11 |
| survival ~ tps(LD1, LD2) + lake + logSL | 2044.120 | 11 |
| survival ~ tps(LD1, LD2) + lake + **treatment** + lake***treatment** + logSL | 2051.536 | 18 |
| survival ~ LD1 + LD2 + lake | 2051.765 | 19 |
| survival ~ tps(LD1, LD2, by: lake) | 2298.805 | 266 |
| survival ~ tps(LD1, LD2, by: **treatment**) | 2993.312 | 960 |
| survival ~ tps(LD1, LD2) | 3034.863 | 1002 |
| log(growth) ~ s(LD1) + s(LD2) + logSL + **treatment** + competitor distance | -3226.596 | - |
| log(growth) ~ tps(LD1, LD2) + s(LD1) + s(LD2) + logSL + **treatment** + competitor distance | -3226.596 | - |
| log(growth) ~ s(LD1) + s(LD2) + logSL + **treatment** | -3214.575 | 12 |
| log(growth) ~ tps(LD1, LD2) + logSL + **treatment** | -3208.659 | 18 |
| log(growth) ~ s(LD1) + s(LD2) + logSL + competitor distance | -3160.196 | 67 |
| log(growth) ~ s(LD1) + s(LD2) + logSL | -3145.952 | 81 |
| log(growth) ~ tps(LD1, LD2) + s(LD1) + s(LD2) + logSL | -3145.952 | 81 |
| log(growth) ~ LD1 + LD2 + logSL | -3131.850 | 95 |
| log(growth) ~ LD1 + LD2 | -2830.445 | 396 |

**Table S7.** Model selection comparison of general additive models for survival and growth rate of hybrids placed in field enclosures in both lakes for the first two major axes of selection (A1 and A2) estimated using generalized projection pursuit projection. The best supported model is indicated first. Notation is adopted from the mgcv package in R: s(A1) indicates a smoothing spline fit to ridge axis one; tps (A1, A2) indicates a thin-plate spline fit to the two ridges; competitor distance indicates the Mahalanobis distance from each hybrid phenotype to the mean phenotype within the 30-trait morphospace while accounting for trait correlations.

| **lake** | **model** | **AIC** | **ΔAIC** |
| --- | --- | --- | --- |
| lake 1 (Crescent Pond) | survival ~ s(A1) + s(A2) | 1819.724 | - |
|  | survival ~ s(A1) + s(A2) + **treatment** | 1820.595 | 0.87 |
|  | survival ~ tps(A1,A2) + **treatment** | 1820.595 | 0.87 |
|  | survival ~ A1 + A2 + **treatment** | 1820.594 | 0.87 |
|  | survival ~ s(A1) + s(A2) + tps(A1,A2) + **treatment** | 1821.649 | 2 |
|  | survival ~ s(A1) + s(A2) + **treatment** + competitor distance | 1822.595 | 3 |
|  | log(growth) ~ s(A1) + s(A1) + logSL + **treatment** + competitor distance | -3230.104 | - |
|  | log(growth) ~ s(A1) + s(A1) + logSL + **treatment** | -3226.259 | 3.8 |
|  | log(growth) ~ s(A1) + s(A1) + logSL + competitor distance | -3185.980 | 44 |
|  | log(growth) ~ s(A1) + s(A1) + logSL | -3180.214 | 50 |
| lake 2 (Little Lake) | survival ~ s(A1) + s(A2) | 108.7232 | - |
|  | survival ~ s(A1) + s(A2) + **treatment** | 110.4928 | 1.8 |
|  | survival ~ s(A1) + s(A2) + tps(A1,A2) + **treatment** | 110.4925 | 1.8 |
|  | survival ~ tps(A1,A2) + **treatment** | 110.4927 | 1.8 |
|  | survival ~ A1 + A2 + **treatment** | 110.4921 | 1.8 |
|  | survival ~ s(A1) + s(A2) + **treatment** + competitor distance | 110.6482 | 1.9 |
|  |  |  |  |

**Table S8.** Proportion of major trait loadings for dominant functional traits along survival fitness pathways leading to lower (orange) or higher (blue) fitness for scale-eater phenotypes in Fig. 6. The proportion is the number of times each trait was the dominant loading on the subsampled discriminant axis out of 1000 random samples of 15 traits out of the 30 total.

|  | **decreasing** | | | | | | **increasing** | | | | | |
| --- | --- | --- | --- | --- | --- | --- | --- | --- | --- | --- | --- | --- |
| **lake** | | **lake 1** | | | **lake 2** | | | **lake 1** | | | **lake 2** | |
| **frequency** | **high** | | **low** | **high** | | **low** | **high** | | **low** | **high** | | **low** |
| lower or upper jaw length | 0.255 | | 0 | 0.569 | | 0.085 | 0.344 | | 0.411 | 0 | | 0.046 |
| nasal protrusion distance or angle | 0.163 | | 0 | 0.080 | | 0.009 | 0.211 | | 0.253 | 0 | | 0.005 |
| adductor height | 0.001 | | 0 | 0.018 | | 0.002 | 0.006 | | 0.002 | 0 | | 0.0002 |
| head height | 0.027 | | 0 | 0.074 | | 0.006 | 0.105 | | 0.097 | 0 | | 0.006 |

**Fig. S1**

**
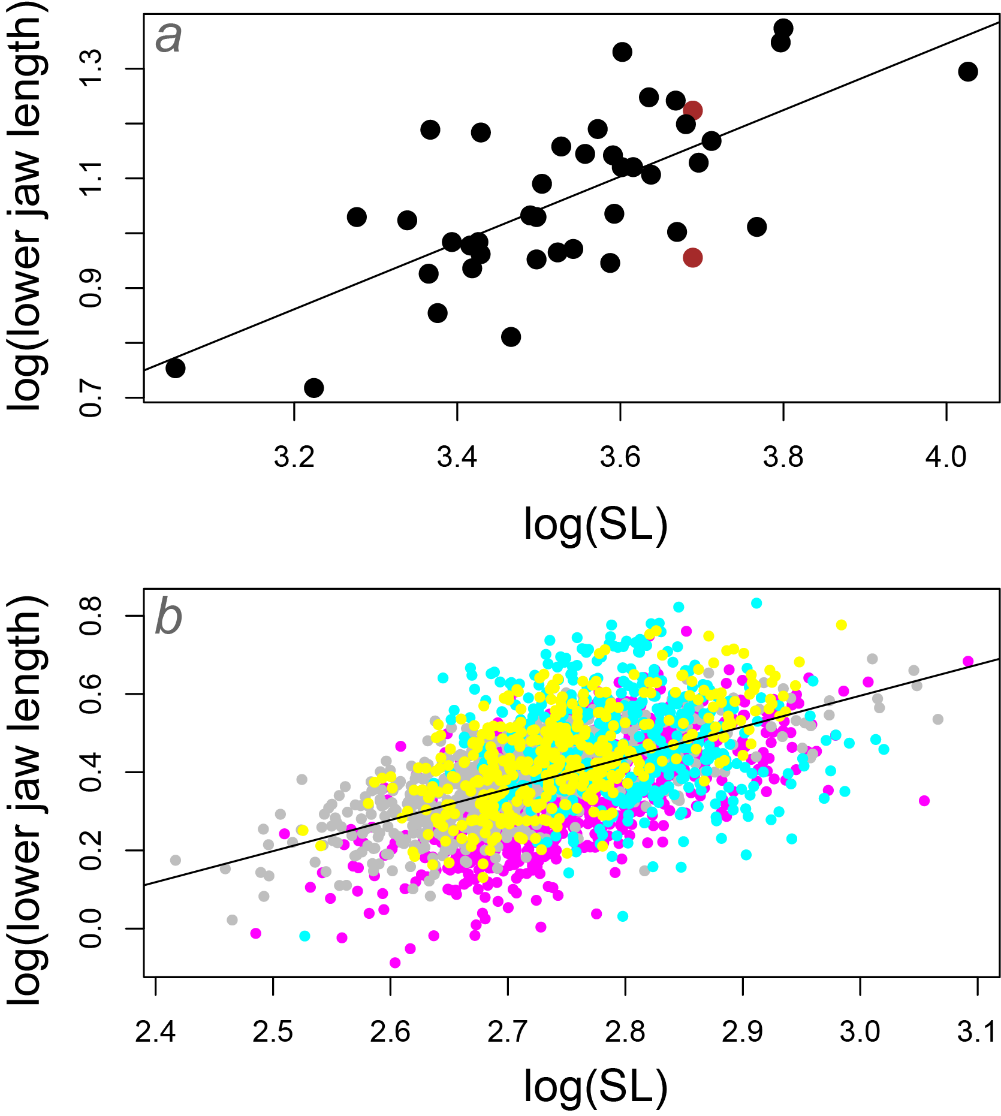
**

**Fig. S1** Log-transformed lower jaw length versus log-transformed standard length for *a)* allopatric Cyprinodontidae species (black) and *b)* hybrid populations (one color per treatment) used in this study. The minimum and maximum upper jaw lengths of allopatric stickleback populations in the Pacific Northwest are also shown for reference (brown). Analyses of data published in (Martin & Wainwright 2011) and Fig. 1 reported in (Lavin & McPhail 1986).

**Fig. S2**

**
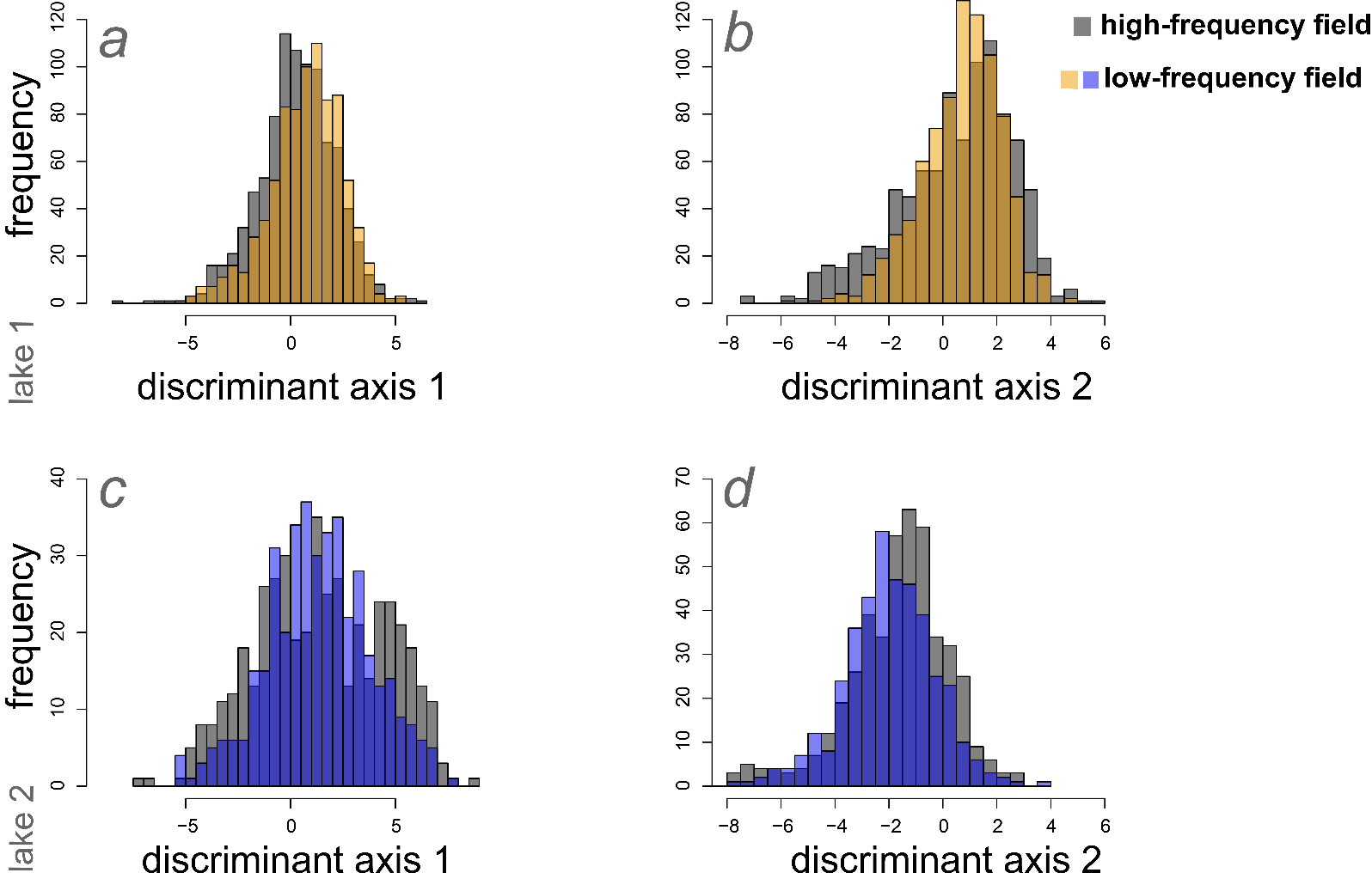
**

**Fig. S2** Histograms depicting the phenotypic variance of hybrid populations in high- (gray bars) and low-frequency (orange/blue) treatments in lake 1 (first row) and lake 2 (second row) on the first and second discriminant axes (LD1 and LD2 from Fig. 3).

**Fig. S3**

**
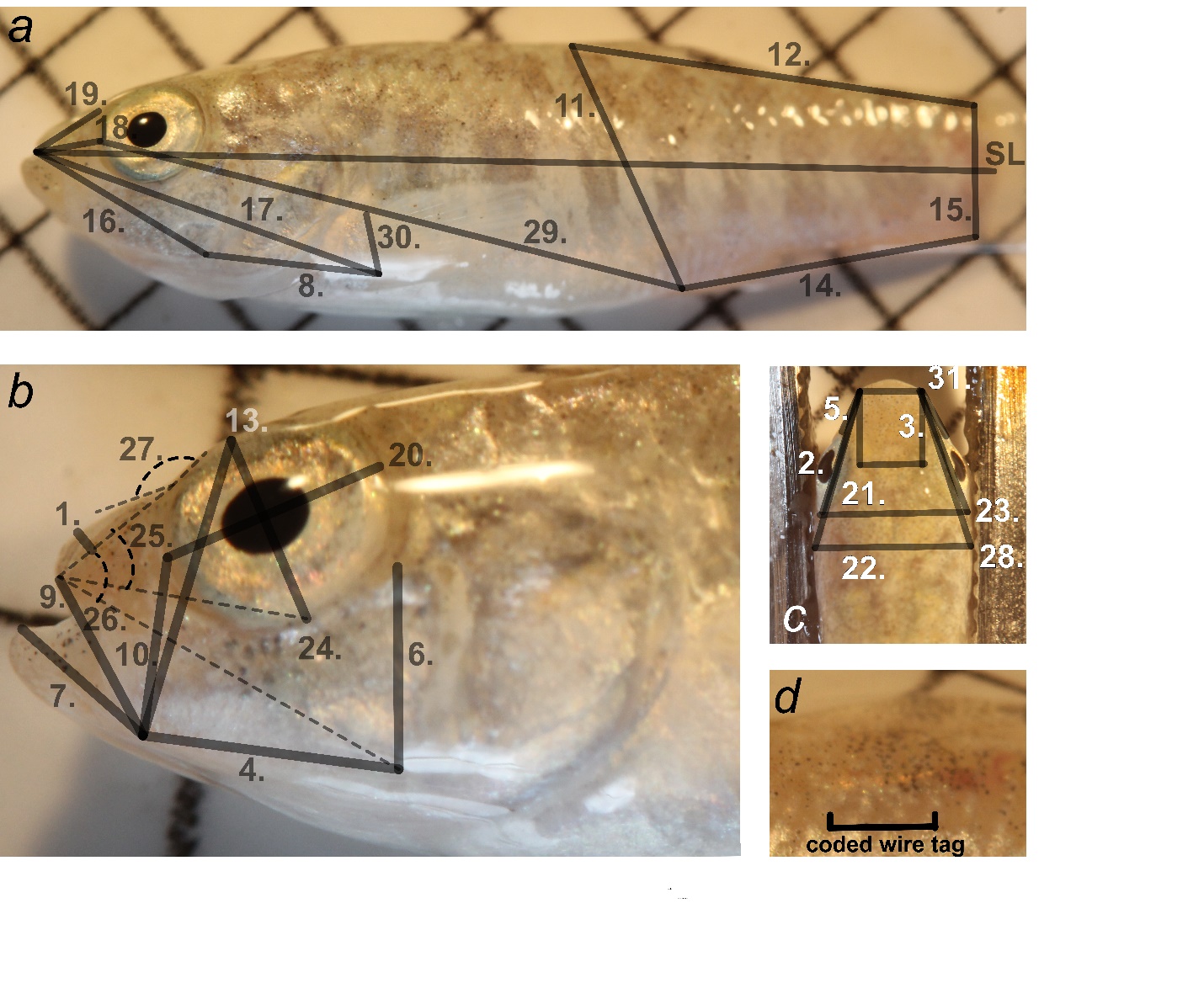
**

**Fig. S3 Morphometric landmarks** indicating the 28 linear distances, 3 angles (25-27), and standard length (SL) for *a)* lateral, *b)* close-up of the craniofacial region, *c)* dorsal view, and *d)* close-up of the injected coded wire tag in the dorsal musculature including injection site (note different hybrid image used here for clarity). Numbers correspond to named traits in Tables 2-4. Lateral measurements were collected from pre-release photographs of both the left and right sides of each hybrid and averaged for all analyses. Dorsal view anteroposterior measurements (2,3,5,23,28) were also measured on each side and averaged for analyses. The 2 mm grid boxes in each image were used for calibration.

**Fig. S4**

**
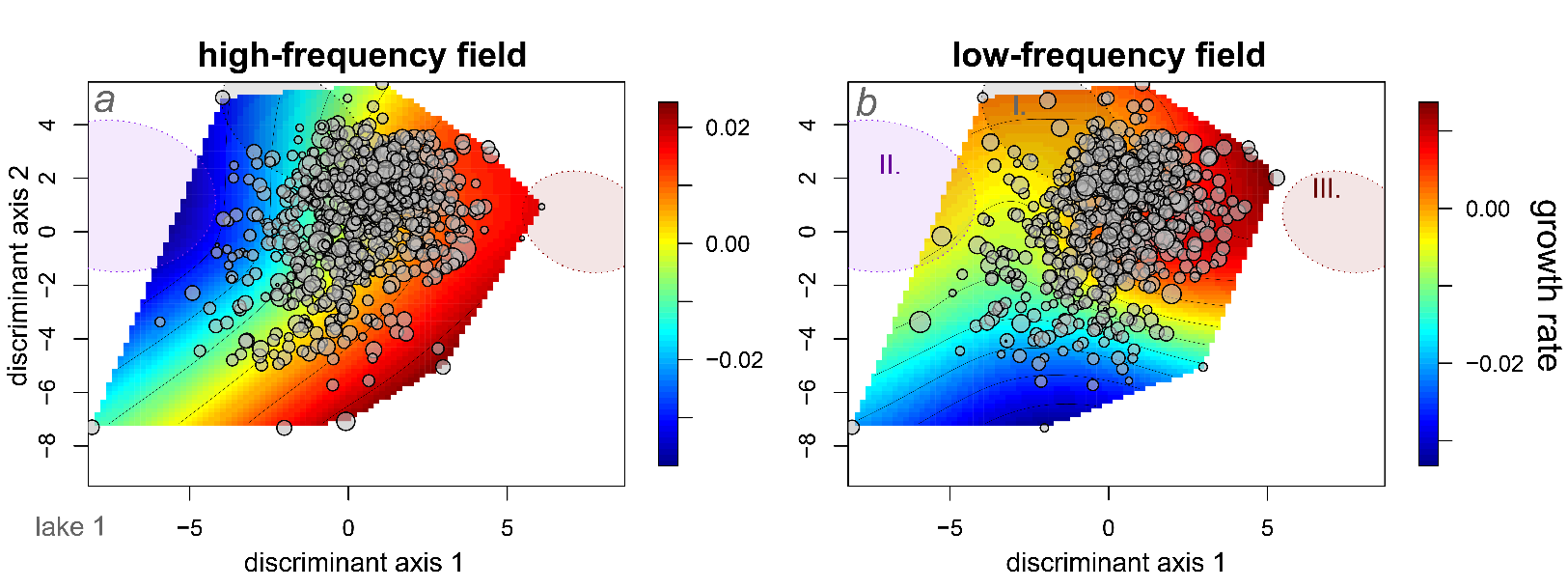
**

**Fig. S4 Growth rate fitness landscapes for a) high-frequency and b) low-frequency treatments in lake 1 (Crescent Pond).** Thin-plate splines predict the growth rate (heat color) across a single linear discriminant morphospace separating generalist and scale-eater phenotypes (x-axis: LD1) and generalist and molluscivore phenotypes (y-axis: LD2). Survivors in field enclosures are depicted in black relative to deaths over the 3-month exposure period. All hybrids are plotted within a shared linear discriminant morphospace calculated from lab-reared F1 individuals of parental populations in both lakes. 95% confidence ellipses indicate generalist (I. grey), molluscivore (II. purple), and scale-eater (III. red) regions of the morphospace.

**Fig. S5**

**
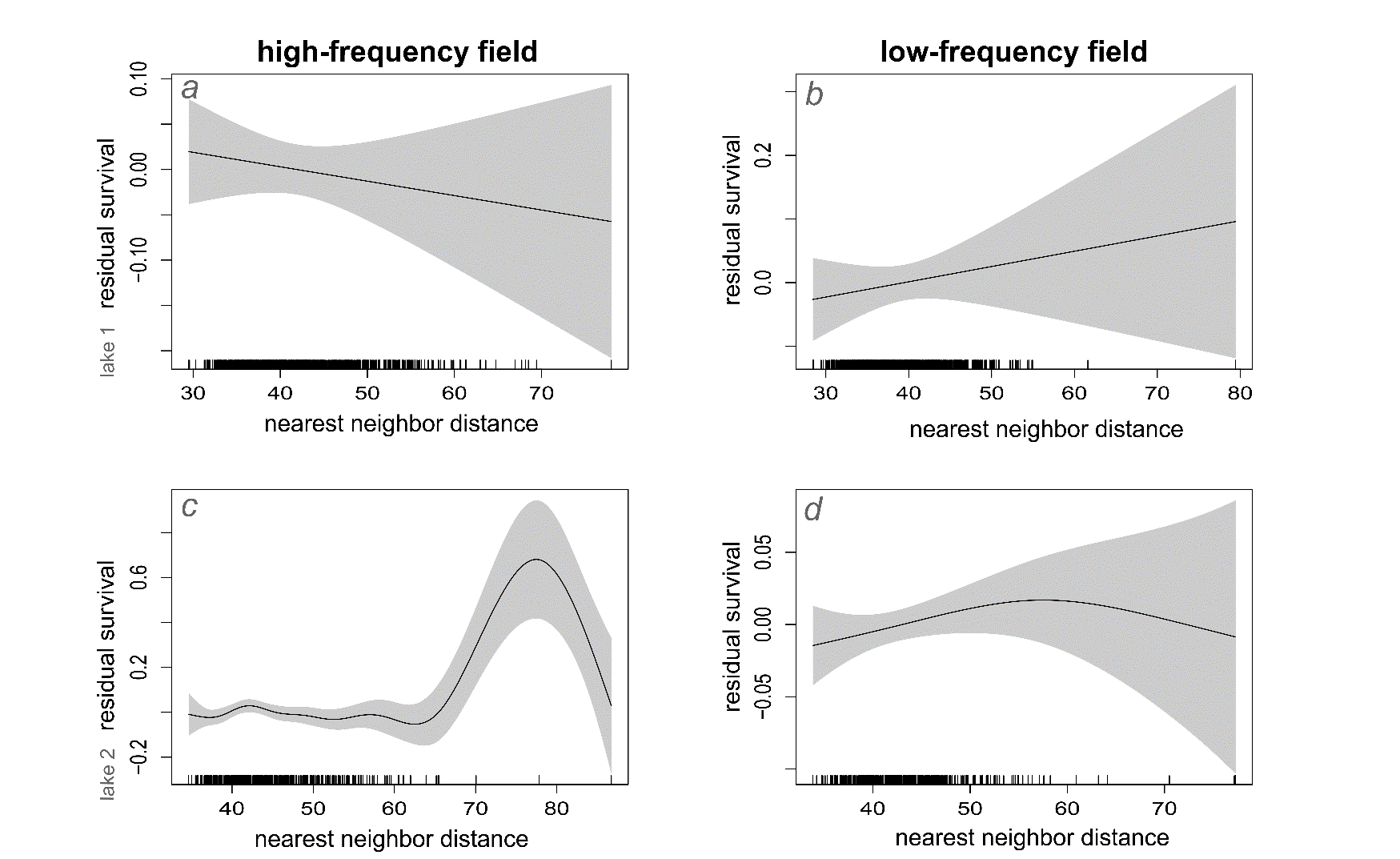
**

**Fig. S5 Residual survival probability relative to the density of similar hybrid phenotypes in high- (first column) and low-frequency (second column) field enclosures.** Residuals calculated from the excess survival probability not explained by the thin-plate splines estimated for hybrid phenotype in the discriminant morphospace (Fig. 3). The frequency of similar hybrid phenotypes was calculated for each hybrid from the nearest-neighbor Euclidean distances to the ten most similar hybrid phenotypes in the full morphospace.

**Fig. S6**

**
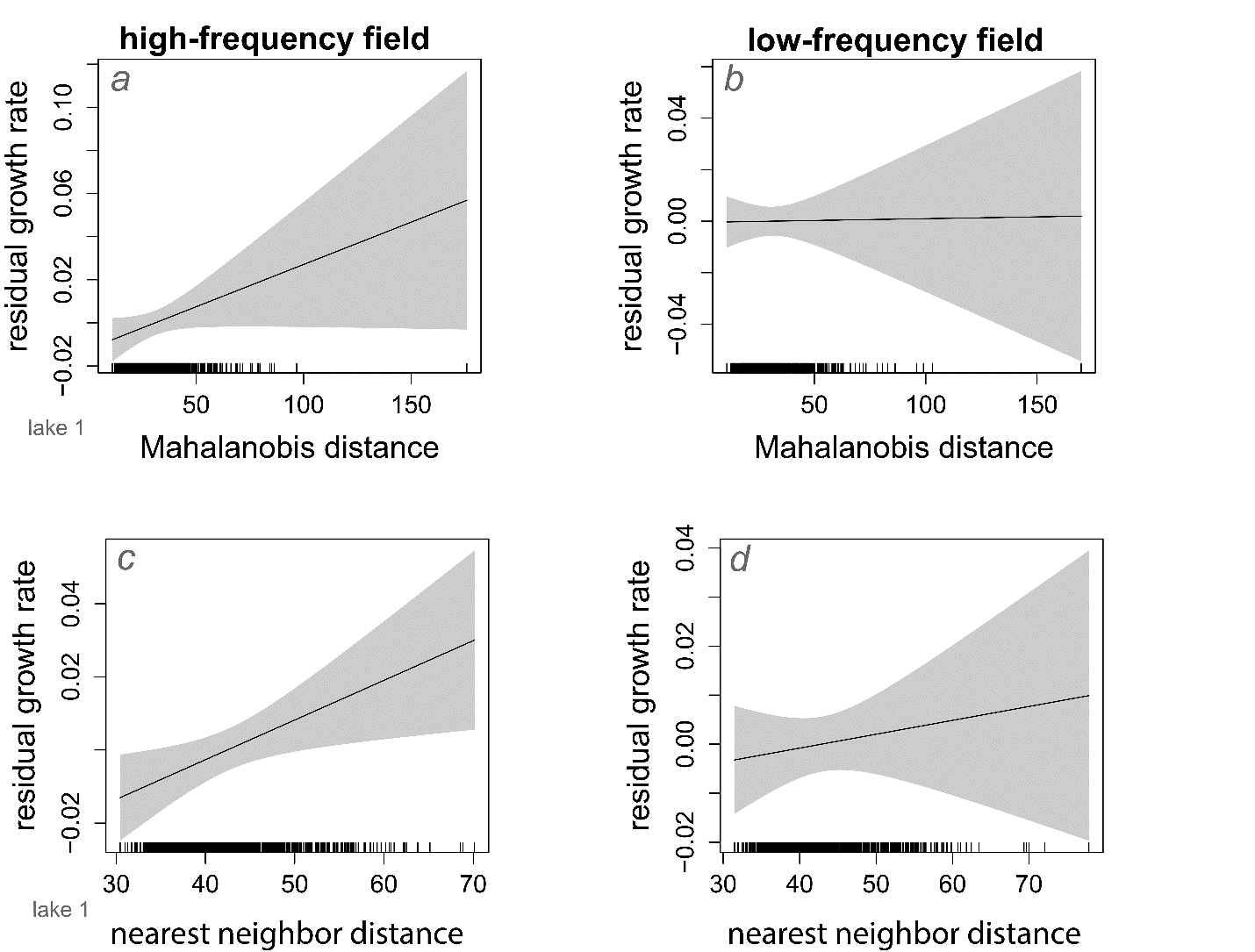
**

**Fig. S6 Residual growth rate relative to the density of similar hybrid phenotypes in high- (first column) and low-frequency (second column) field enclosures.** Residuals calculated from the growth rate variance not explained by the thin-plate splines estimated for hybrid phenotype in the linear discriminant morphospace (Fig. S5). The frequency of similar hybrid phenotypes was calculated for each hybrid from *a-b)* the Mahalanobis distance to the mean hybrid phenotype within each treatment and *c-d)* the sum of nearest-neighbor Euclidean distances to the ten most similar hybrid phenotypes in the full 30-trait morphospace.

**Fig. S7**

**
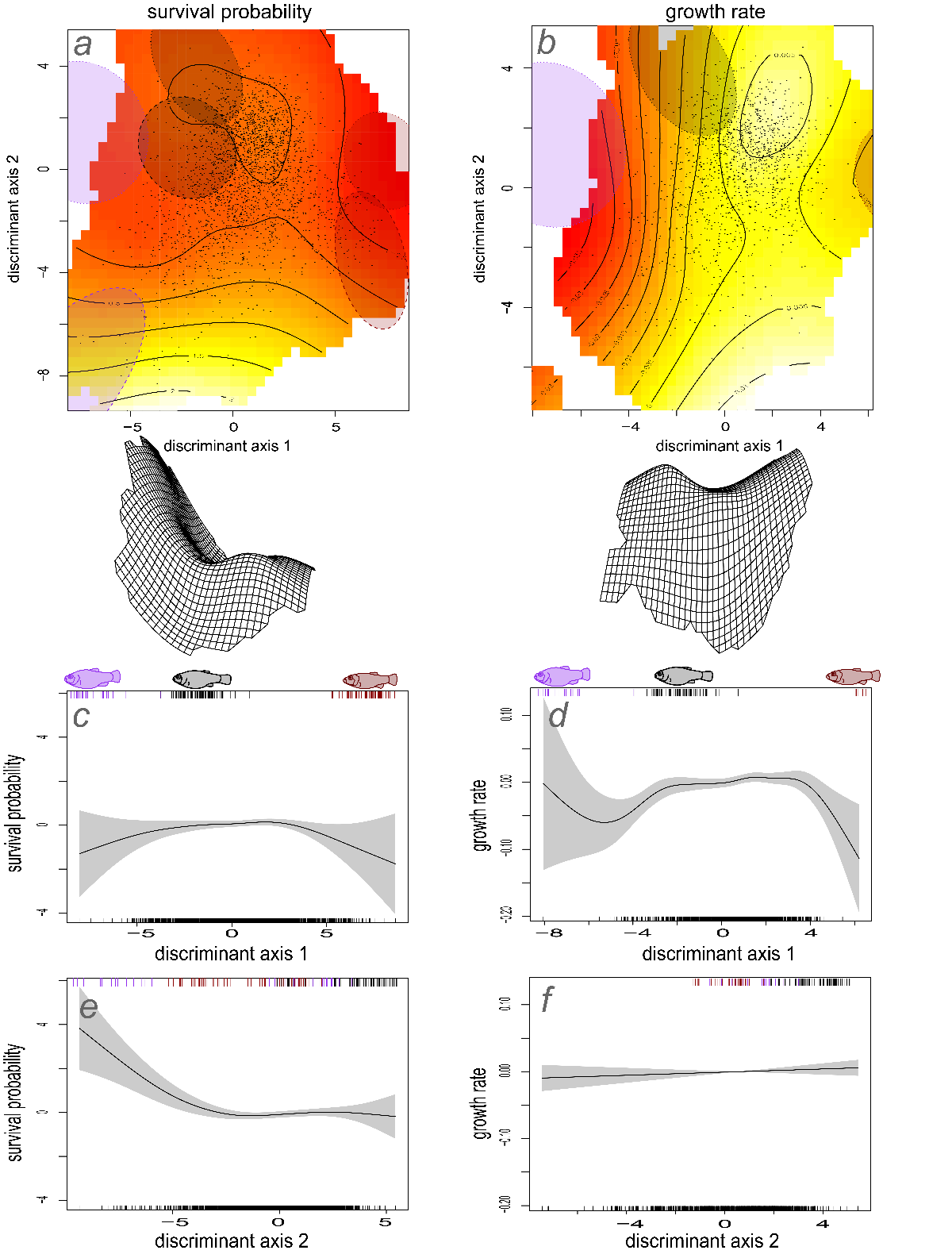
**

**Fig. S7 Joint survival (first column) and growth (second column) fitness landscapes estimated across treatments and lake environments using generalized additive modeling.** Thin-plate splines estimate the probability of *a)* survival controlling for lake and treatment effects across all four enclosures and *b)* growth rate across both enclosures in lake 1 (lake 2 was excluded due to low survival rates). Thin-plate splines and smoothing splines (± 1 SE) are depicted within the linear discriminant morphospace separating generalist and scale-eater phenotypes (x-axis: LD1) and generalist and molluscivore phenotypes (y-axis: LD2) calculated from laboratory-reared individuals of parental populations in both lakes. 95% confidence ellipses show the location of generalist (grey), molluscivore (purple), and scale-eater (red) parental populations from lake 1 (small dashed line) and lake 2 (large dashed line). All hybrids are represented by points or tick marks on the x-axis and parental individuals are represented by tick marks on the upper margin.

**Fig. S8**

**
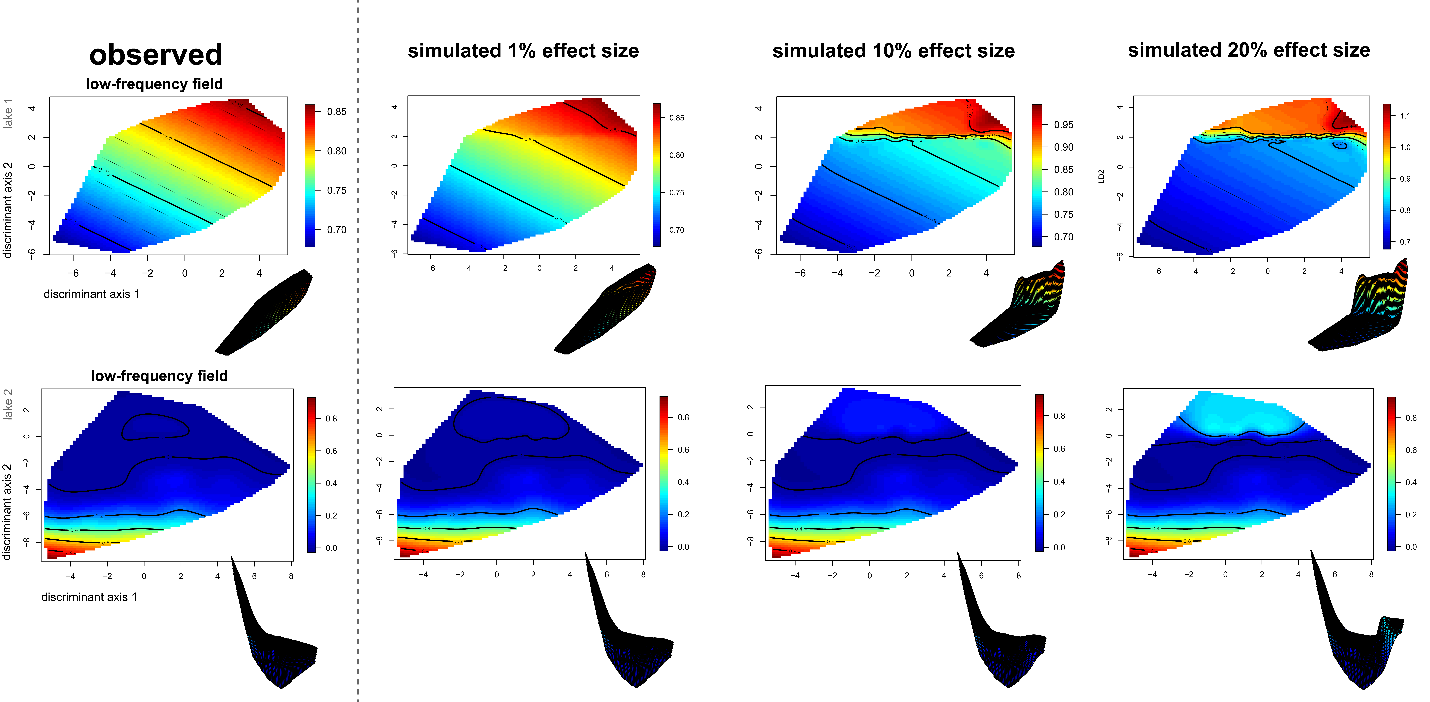
**

**Fig. S8 Simulated 1%, 10%, and 20% effect sizes in the upper region (top fifth) of the survival fitness landscapes for both low-frequency field enclosures.** 3D insets show the increasing nonlinear curvature at the edge of the discriminant morphospace, comparable to the negative frequency-dependent disruptive selection predicted by competitive speciation theory. The power of our generalized additive models to detect these true effect sizes was measured by counting the number of times the frequency term in our models was significant at an alpha = 0.05 threshold in 1,000 binomial samples from these simulated surfaces, holding our observed sample sizes and phenotypic distributions constant. For example, each random sample from the simulated 1% effect size surfaces drew a new set of 833 binomial samples from lake 1 and 404 binomial samples from lake 2 (the number of individuals measured in each low-frequency enclosure) based on the survival probability depicted in this figure. This random sample drawn under a true effect size of a 1% increase in survival in one region of the morphospace was then compared to the observed high-frequency fitness landscapes using our GAM models to determine if the frequency effect detected the true difference (survival ~ tps(LD1, LD2) + treatment; or survival ~ s(LD1) + s(LD2) + tps(LD1, LD2) + treatment).
